# The influence of speed and size on avian terrestrial locomotor biomechanics: Predicting locomotion in extinct theropod dinosaurs

**DOI:** 10.1371/journal.pone.0192172

**Published:** 2018-02-21

**Authors:** P. J. Bishop, D. F. Graham, L. P. Lamas, J. R. Hutchinson, J. Rubenson, J. A. Hancock, R. S. Wilson, S. A. Hocknull, R. S. Barrett, D. G. Lloyd, C. J. Clemente

**Affiliations:** 1 Geosciences Program, Queensland Museum, Brisbane, Queensland, Australia; 2 School of Allied Health Sciences, Griffith University, Gold Coast, Queensland, Australia; 3 Innovations in Health Technology, Menzies Health Institute Queensland, Gold Coast, Queensland, Australia; 4 Structure and Motion Laboratory, Department of Comparative Biomedical Sciences, Royal Veterinary College, Hatfield, Hertfordshire, United Kingdom; 5 Faculdade de Medicina Veterinária, Universidade de Lisboa, Lisbon, Portugal; 6 Biomechanics Laboratory, Department of Kinesiology, Pennsylvania State University, University Park, Pennsylvania, United States of America; 7 School of Human Sciences, University of Western Australia, Crawley, Australia; 8 Murphy Deming College of Health Sciences, Mary Baldwin University, Staunton, Virginia, United States of America; 9 School of Biological Sciences, The University of Queensland, Brisbane, Queensland, Australia; 10 School of Science and Engineering, University of the Sunshine Coast, Maroochydore, Queensland, Australia; Perot Museum of Nature and Science, UNITED STATES

## Abstract

How extinct, non-avian theropod dinosaurs moved is a subject of considerable interest and controversy. A better understanding of non-avian theropod locomotion can be achieved by better understanding terrestrial locomotor biomechanics in their modern descendants, birds. Despite much research on the subject, avian terrestrial locomotion remains little explored in regards to how kinematic and kinetic factors vary together with speed and body size. Here, terrestrial locomotion was investigated in twelve species of ground-dwelling bird, spanning a 1,780-fold range in body mass, across almost their entire speed range. Particular attention was devoted to the ground reaction force (GRF), the force that the feet exert upon the ground. Comparable data for the only other extant obligate, striding biped, humans, were also collected and studied. In birds, all kinematic and kinetic parameters examined changed continuously with increasing speed, while in humans all but one of those same parameters changed abruptly at the walk-run transition. This result supports previous studies that show birds to have a highly continuous locomotor repertoire compared to humans, where discrete ‘walking’ and ‘running’ gaits are not easily distinguished based on kinematic patterns alone. The influences of speed and body size on kinematic and kinetic factors in birds are developed into a set of predictive relationships that may be applied to extinct, non-avian theropods. The resulting predictive model is able to explain 79–93% of the observed variation in kinematics and 69–83% of the observed variation in GRFs, and also performs well in extrapolation tests. However, this study also found that the location of the whole-body centre of mass may exert an important influence on the nature of the GRF, and hence some caution is warranted, in lieu of further investigation.

## Introduction

What would an eight tonne *Tyrannosaurus rex* have looked like moving at 5 m s^-1^? Could it have managed to move as fast as 10 m s^-1^, or faster? A perennial question of interest for palaeontologists is how extinct animals appeared and behaved when they were alive. Perhaps more than any other animal group, theropod dinosaurs have received considerable attention as to how they stood and moved, owing to their evidently carnivorous lifestyle and the gigantic sizes that many species attained, despite their bipedal stance [1]. In particular, lively debate has persisted for decades as to the athletic capabilities of the largest theropods, especially the non-avian species such as *Tyrannosaurus*, *Giganotosaurus* and *Allosaurus* [2–11].

One way of better understanding how extinct theropods moved is to examine locomotion in extant theropods, birds, because birds retain many (homologous) anatomical similarities to their ancestors [12–18]. Additionally, humans, as the only other extant obligate, striding biped, also serve as an important source of information concerning bipedal locomotion in extinct theropods. By identifying and understanding the similarities and differences between bird and human locomotion, it is possible to elucidate fundamental aspects of obligate striding bipedalism, and those that are influenced by anatomical or postural differences.

Previous studies have indicated that terrestrial locomotion in modern birds is considerably different from that in humans. Birds employ a crouched, digitigrade, parasagittal posture, whereby the femur is subhorizontally oriented for much of the stride, and where the majority of limb movement occurs at the knee, driven by the ‘hamstring’ muscles [19–33]. This reflects the location of their whole-body centre of mass (COM), which is markedly anterior to the hips [31,34]. The gaits of birds also appear to be less discrete than those of humans, because birds employ a ‘grounded run’ at intermediate speeds of locomotion [21,22,28,30,35–39]. During grounded running, the whole-body COM oscillates with little exchange of kinetic and potential energies (i.e., kinetic and potential energies oscillate out of phase; characteristic of human running), yet duty factors are greater than 0.5 (no aerial phase; characteristic of human walking). Only at greater speeds do duty factors decrease below 0.5, resulting in running with an aerial phase. Thus, while ‘walking’ and ‘running’ are easily recognizable in humans, the distinction is less obvious in birds.

One aspect of avian terrestrial locomotion that remains less explored, in comparison to humans, is how kinematics and kinetics change together with increasing speed, or how these are influenced by body size (especially in a comparative context). The potential influence of body size is particularly pertinent, given the large sizes that many extinct theropods attained, greater than those observed in extant species. The effect of speed on kinematic parameters have been investigated many times previously [19,22,28,30,36–38,40–42]; yet, starting with the seminal work of Gatesy and Biewener [21], only a handful of studies have examined speed effects across species of different body size [24,29,32,43]. Of the few studies that have examined speed effects on kinetic parameters [28,30,37,44–47], only three have investigated more than a single species [45–47]. Even then, only mechanical energy fluctuations were examined, whilst many other kinetic aspects such as forces (external or internal), joint moments or powers remain to be explored. Consequently, there is a need for further exploration and integration of how the fundamental aspects of terrestrial locomotion are modulated by speed and body size in ground-dwelling birds. Without a better understanding of speed or size effects on locomotion, this hampers attempts to quantitatively characterize locomotion in extinct theropods, through empirical extrapolation or theoretical modelling [e.g., 5–7,10,48,49,50]

The present study builds upon previous research in an attempt to better understand locomotor biomechanics in both extant and extinct theropods. Its primary objective is to examine how several basic kinematic and kinetic variables of terrestrial locomotion vary with speed and body size in modern ground-dwelling birds, with the view to deriving predictive relationships that can be applied to extinct theropods. The ability to better predict these variables for extinct theropods will facilitate improved attempts at understanding their locomotor behaviour through biomechanical modelling (e.g., [5–7,10,11,48,50]). A key feature of interest is the ground reaction force (GRF), the force that the feet exert upon the ground during locomotion. As a reaction force, it represents the summation of accelerations of all the individual components of the body during movement, and is fundamental to understanding the forces, moments and stresses occurring within the limbs of an animal during locomotion [7,35,51–56]. Despite its importance, the GRF has been measured in relatively few species of birds, in the course of investigating other biomechanical parameters [26,30,33,35,37,40,46,53,57–61]. Hence, not enough is currently known about how the GRF varies in time and space throughout the stance phase in different bird species, or how this varies with speed or body size, to the point that quantitative predictions may be made for other theropods (avian or non-avian).

This study also aimed to further compare locomotion between birds and humans, building upon previous work such as that of Gatesy and Biewener [21], to better elucidate the underlying similarities and salient differences between the two groups. In addition to better characterizing avian terrestrial locomotion, this can help identify those aspects of obligate striding bipedalism that are influenced by anatomical or postural differences. In turn, this will provide guidance on the use of any predictive relationships derived herein.

## Methods

A summary of the methodology employed in this study is given below; a detailed outline is presented in the Supporting Information (S1 Appendix). Two data sets were collected: three-dimensional (3-D) kinematic and kinetic data for modern ground-dwelling bird species and 3-D kinetic data for humans. All experimental protocols used in this study were approved by the Griffith University and University of Queensland research ethics committees (approvals AHS/01/14/AEC, SBS/102/14/ARC). Collection of the three wild bird species was approved by the Queensland Department of Environment and Heritage Protection (permit WISP14699514). Written informed consent was obtained from the human subjects prior to the study.

Since the distinction between ‘walking’ and ‘running’ is not often clear in birds (as noted above), the use of these terms for describing gait is herein restricted to the observations for humans, where ‘walking’ and ‘running’ are easily distinguished, by either kinematic or kinetic criteria. No attempt is made to define gaits for the birds studied; the observations are reported and interpreted here in terms of speed, rather than a particular gait.

### Bird data

#### Animals studied

Twelve species of ground-dwelling bird were studied (Table 1), ranging from 45 g Chinese painted quail to 80 kg ostriches. Only ground-dwelling species were investigated here because, by virtue of spending most or all of their time on the ground, they have well-developed hindlimb locomotor systems. Data for tinamous, ostriches and emus had been collected in previous studies (tinamous: [37]; ostriches: [25,60]; emus: [62]). Given logistical limitations and the objectives of this study, preference was given to maximizing the diversity of bird species investigated, rather than achieving a greater number of replicates for fewer species. All birds investigated in the present study were considered to be adults based on skeletal maturity, with the possible exception of the domestic turkeys, which had sizeable chondroepiphyses (as revealed in postmortem dissections), but were of adult size. Kinematic data for all species were collected using 3-D motion capture techniques, except that for the tinamous where only a single camera was used, placed perpendicular to the direction of travel [37]. GRF data was collected in three dimensions for all species.

**Table 1:** The species of bird studied. Reported are sample sizes and mean (± s.d.) masses, hip heights and total leg lengths (sum of interarticular lengths of femur, tibiotarsus and tarsometatarsus).

Species		*n*	Mass, kg	Hip height, mm	Leg length, mm	Data collected
scientific name	common name					
*Coturnix chinensis*	Chinese painted quail	3♂, 2♀	0.047 ± 0.002	58.6 ± 4	84 ± 3.2	this study
*Colinus virginianus*	Northern bobwhite quail	3♂, 2♀	0.17 ± 0.014	77.8 ± 10.4	133.2 ± 2.8	this study
*Coturnix japonica*	Japanese quail	1♂, 3♀	0.301 ± 0.077	106.3 ± 7.5	140.5 ± 8.7	this study
*Porphyrio porphyrio*	Purple swamphen	3♀	0.623 ± 0.058	239 ± 14.1	287.7 ± 9.1	this study
*Eudromia elegans*	Elegant-crested tinamou	3	0.756 ± 0.013	172 ± 6.7	n/a	ref. [37]
*Numida meleagris*	Helmeted guineafowl	2♂, 1♀	1.257 ± 0.114	201.7 ± 15.5	249.3 ± 8.3	this study
*Alectura lathami*	Australian brush turkey	2♀	1.49 ± 0.057	267 ± 15.6	309.5 ± 2.1	this study
*Threskiornis moluccus*	Australian white ibis	2♂	1.54 ± 0.057	282.5 ± 30.4	342 ± 15.6	this study
*Gallus gallus*	Domestic chicken (white leghorn breed)	1♂, 2♀	1.71 ± 0.521	254.3 ± 47.8	284.3 ± 43.8	this study
*Meleagris gallopavo*	Domestic turkey (various mixed breeds)	2♂, 3♀	3.228 ± 0.9	365.2 ± 47.4	406.8 ±49.3	this study
*Dromaius novaehollandiae*	Emu	6♀	38.58 ± 2.69	903.3 ± 35.0	1005.5 ± 25.9	ref. [62]
*Struthio camelus*	Ostrich	3♂	74.35 ± 6.15	1128 ± 14.1	1181 ± 0	refs [25,60]

### Data collection

In the present study, a small indoor racetrack was used for the three quail species, and a larger outdoor racetrack was used for the remaining species. Both racetracks were walled around their entire perimeter, but the middle of one side was replaced with either clear acrylic or fine wire mesh, through which filming of the birds took place. Birds were filmed at 50–250 frames second^-1^ with two high-speed light video cameras (IL3-100 and HiSpec1, Fastec Imaging Corporation, San Diego, California, USA), synchronized using a manual trigger pulse. For both racetracks, a calibration volume for each day’s trials was established using an 11-coefficient direct linear transform [63].

Custom-built forceplates were mounted in the middle of both racetracks; both were covered with fine grit sandpaper and flush with the surrounding racetrack surface. In the small racetrack, the forceplate comprised a plastic top plate secured to a six degree-of-freedom force-torque sensor (ATI nano17; ATI Industrial Automation, Apex, North Carolina, USA). In the large racetrack, one of two forceplates was used. The first one, used for the majority of data collection, has been described in detail elsewhere [64]. The second forceplate, used in the collection of a portion of the guineafowl data, comprised a plastic top plate secured to a six degree-of-freedom force-torque sensor (ATI gamma; ATI Industrial Automation). The forceplates were operated through an analogue-digital converter (NI USB-6343, National Instruments, Austin, Texas, USA), which sampled data at 10 kHz using a custom LabVIEW 2012 script (National Instruments). Cameras were synchronized with each other and the forceplates using a manual trigger pulse.

Prior to data collection, feathers from the back half of the birds were clipped, as were the wings, so as to allow the placement of small (2–5 mm diameter) markers, and so that the cameras’ views of the markers during locomotion was not obstructed. Up to three markers were placed on the midline of the back, along the sacrum. A single marker was placed over the trochanteric crest of each hip, which in all species was easily palpable. The base of the claw of digit III of both feet was also marked with non-toxic, white acrylic paint.

Birds moved down the racetrack and over the forceplate on their own accord at a self-selected speed, although sometimes additional motivation was used, such as making loud noises. A wide range of speeds was studied; the slowest incorporating substantial periods of double limb support and the fastest involving substantial aerial phases. Birds were also filmed during stationary standing, allowing the capture and measurement of the height of the hip marker on the birds, which was taken as standing hip height. After concluding the experiments, the birds were euthanased by cervical dislocation and immediately massed. The right leg was then dissected free of soft tissue and the interarticular lengths of the femur, tibiotarsus and tarsometatarsus measured (excluding trochanters or crests). The sum of these lengths is taken to be the total leg length, *L*.

### Kinematic data processing

The back and toe markers were digitized and their 3-D coordinates calculated using DLTdv5 [63], a program written for MATLAB (version 8.0, MathWorks, Natick, Massachusetts, USA). From the digitized marker trajectories four kinematic parameters were determined: mean forward speed (*v*), stance duration (*t*_stance_), stride length (*S*) and duty factor (*β*, the proportion of the stride for which a given foot is on the ground). In some instances, the vertical component of the GRF was also used in the calculation of *β*, by identifying the onset and offset of vertical force above the background level of noise. In standing trials, the hip marker was digitized and its coordinates calculated, either using DLTdv5 or using a reference object of known dimensions within the field of view of the cameras. The calculated *z*-coordinate of the hip marker was taken as the standing hip height *h* of the bird. For each trial, speed, stance duration and stride length were normalized to the standing hip height of the individual bird, as follows:

$$v*=\frac{v}{\sqrt{gh}},$$ (1)

$$t_{\mathrm{stance}}^{}*=t_{\mathrm{stance}}\cdot \sqrt{\frac{g}{h}},$$ (2)

S*=S/h, (3)

where *g* is the acceleration due to gravity, 9.81 m s^-2^. As defined here, relative speed v* is equal to the square root of the Froude number (‘dimensionless speed’) as often used in other studies (e.g., [65,66]).

#### GRF data processing

The raw forceplate data were processed and converted to Newtons using a set of custom MATLAB scripts [64]. Each channel was filtered with a fourth-order, zero-lag Butterworth low-pass filter; the cutoff frequencies used removed noise without attenuating signal peaks, and were chosen based on visual comparison of the filtered *versus* unfiltered data. These varied between 15–100 Hz, depending on the forceplate used and species in question. The ostrich force data had previously been filtered at 15 Hz [60]. Lastly, the measured forces were corrected for the direction of heading of the bird and the foot (left or right) that contacted the forceplate, so as to express them in a consistent anatomical coordinate system: +*x* is forward, +*y* is medial and +*z* is upwards.

### Human data

#### Data collection

Three healthy, adult, recreationally active volunteers (two male, one female) were studied (height 179.3 ± 3.2 cm, mass 79.7 ± 16.8 kg, means ± s.d.). A sample size of three was deemed sufficient, since the aim of the study was to elucidate major patterns, for broad comparative purposes [21,60,65–67]. The subjects walked and ran barefoot along a split-belt instrumented treadmill that recorded three-dimensional GRFs at 1 kHz (Bertec Limited, Columbus, Ohio, USA). The speed of the treadmill was controlled via external computer software. Each subject undertook a number of walking and running trials at speed increments of 0.25 m s^-1^, ranging from slow walking to fast running speeds. Subjects were also tested twice at their (predetermined) walk-run transition speed, in one trial using walking, and in the other using running [43].

#### Data processing

Although both belts of the instrumented treadmill recorded GRFs, only the GRFs of the right footfalls for each trial were extracted for further processing. The instances of foot touchdown and liftoff for each footfall were determined from the raw force data. The force data for each footfall was then filtered with a fourth-order, zero-lag Butterworth low-pass filter, with cutoff frequencies largely between 15–25 Hz. The instances of touchdown and liftoff also enabled the determination of *t*_stance_, *β* and *S*, the latter calculated as

$$S=v\cdot t_{\mathrm{stride}},$$ (4)

where *t*_stride_ is the stride duration. As for the birds, *v*, *t*_stance_ and *S* were normalized using Eqs (1–3), where each subject’s *h* was determined from standard anthropometry [68], based on the subject’s total standing height.

### GRF analysis

For each bird and human trial, the processed 3-D GRF profile was analyzed in a custom MATLAB script to examine its more salient aspects. This determined the following parameters: the magnitude of peak net GRF; the magnitude of peak vertical, anteroposterior (positive and negative) and mediolateral components of the GRF; the timing of these peaks; the mean magnitude of the vertical component of the GRF over the stance; the magnitude of the GRF and its components at midstance; and the magnitude of the vertical force when the anteroposterior force was zero (i.e., the magnitude of the vertical force when the GRF was vertical in the sagittal plane). Additionally, the GRF was used to investigate fluctuations of kinetic and potential energy (KE and PE, respectively) of the whole-body COM. Usually, the GRFs from an entire stride (or integral number of strides) are required to examine patterns of mechanical energy fluctuation, through single and double integrations of the force-time profiles [37,46,47,69,70]. However, as *β* was known, this was able to be achieved by superimposing the recorded single-footfall GRF profile onto itself (with an appropriate temporal offset) to simulate the total, whole-stride GRF profile experienced throughout the course of a whole stride (see S1 Appendix for full details).

Once KE and PE were calculated across the entire stride, an assessment of their fluctuation relative to each other was made, by calculating percent congruity [71]. This is a measure of how often PE and KE fluctuate in phase; that is, how often the slopes of the KE-time and PE-time curves have the same sign. In pure vaulting mechanics, percent congruity is 0%, and in pure bouncing mechanics percent congruity is 100%. In addition to percent congruity, the net vertical displacement (NVD) of the COM was determined, calculated as the difference between the maximum and minimum values of the instantaneous vertical displacement of the whole-body COM. When normalized to hip height, NVD therefore expresses how much the COM moves up and down throughout the stride.

As a key objective of this study is prediction of the GRF, the force-time profile of each of the three components of the GRF (normalized to body weight and stance duration) were also subjected to Fourier analysis to determine the corresponding Fourier series, implemented in a custom MATLAB script. A Fourier series is a summation of first-order sine and cosine terms of increasing frequency,

$$f(t)=a_{0}+\sum_{n=1}^{\infty }\left(a_{n}\sin(\pi nt)+b_{n}\cos(\pi nt)\right),$$ (5)

where the pronumeral *n* refers to the fundamental frequencies (1 Hz, 2 Hz, 3 Hz, 4 Hz,…). Given enough terms (high enough *n*), the coefficients *a*_*n*_ and *b*_*n*_ of a Fourier series can replicate the shape of *any* waveform. Thus, Fourier series are an attractive means of concisely describing the entire shape of a force profile (see also [72,73]), effectively obviating the need to measure individual quantities such as peak vertical force.

Prior to the analyses outlined above, the vertical component of the GRF profile was corrected for dynamic inconsistency between it and measured kinematics for each trial. The logic behind this is outlined as follows. In an ideal situation of steady-state locomotion, over an integral number of strides the net change in vertical position of the COM is zero, as is the net change in vertical velocity and vertical momentum. For vertical momentum to exhibit no net change across an integral number of strides, this means, by the law of conservation of momentum, that the net vertical impulse is zero; so for a biped,

$$2I_{z}=I_{w}.$$ (6)

That is, the upward impulse from the foot contacting the forceplate, *I*_*z*_, should theoretically equal half of the downward impulse of the body's weight over the stride, *I*_*w*_, or equivalently, the impulse of the body’s weight over half the stride [74]. This is equivalent to stating that the mean vertical force exerted by the feet over one stride is equal and opposite to body weight [75,76]. However, this is not always the case, because of errors in measurement of mass, stance duration, duty factor or force, which lead to dynamic inconsistency in measured kinematics and kinetics. There may also be unevenness in the distribution of body weight across the two feet, although this is assumed to be negligible here as the trials studied are of straight line locomotion (but see [45], where asymmetric force production was reported). Akin to residual reduction analysis [77], the dynamic inconsistency can be remedied by adjusting the vertical component of the GRF to match the expected impulse. The simplest means of achieving this is a linear (proportional) scaling of the magnitude of the force at each time instance,

$$F_{z(\mathrm{adjusted})}=\alpha \cdot F_{z}.$$ (7)

The adjustment factor *α* is the ratio of half of the body weight impulse to the impulse of the recorded *F*_*z*_:

$$\alpha =\frac{m\cdot g\cdot t_{\mathrm{stance}}}{2\beta \cdot \int_{0}^{t_{\mathrm{stance}}}F_{Z}dt},$$ (8)

where *m* is body mass. The closer the value of *α* is to unity the greater dynamic consistency exists between recorded kinematics and kinetics. Moreover, as it is assumed that asymmetry in force distribution across the feet is negligible, the adjustment is only applied to the vertical component of the GRF.

### Statistical analyses

All analyses were conducted in R 3.2.2 [78], PAST [79] or MATLAB. As the primary objective of this study was to predict locomotor kinematics and kinetics based on body size and speed, two main sets of statistical analysis were conducted. Firstly, allometric relationships between the morphometric variables of total leg length *L*, hip height *h* and body mass *m* were investigated in the birds. In this study, all kinematic and kinetic parameters were normalized by either *h* or *m*, as is typical in comparative biomechanical studies. However, neither variable is known for an extinct theropod, but they can be estimated. Hip height is often estimated from fossil footprints (e.g., [80,81], but see below), although it is very difficult to definitively associate a set of footprints with a particular species. Body mass can be estimated in several ways (e.g., [82–87]), although estimates can have considerable margins of error. One variable that can be measured directly from an extinct theropod’s skeleton is *L* (minus articular cartilage, which normally does not fossilize). Hence, ascertaining the relationships between *L*, *h* and *m* can be useful for predicting morphometrics in extinct theropods. In addition to direct comparisons between these three variables, a fourth derived variable was also investigated, this being a dimensionless measure of the ‘degree of crouch’, which was defined as

$$DC=1-\frac{h}{L}.$$ (9)

Thus, a bird that stands with a more erect limb posture will have a lower *DC* value.

The second set of statistical analyses examined the effect of speed on kinematic and kinetic parameters, the latter including features directly measurable from the GRF force-time profiles, as well as Fourier coefficients and measures of mechanical energy fluctuation (detailed above). As the central aim of this study was prediction, ordinary least squares regressions were used throughout [88], unless otherwise specified. Moreover, given this objective, it was imperative to maximize the generality of the predictive equations derived, even if this entailed sacrificing some fine-scale accuracy concerning the effects of body mass or phylogeny.

Three types of predictive equation were considered for each variable: a linear fit (*y* = *Ax* + *B*), a simple power fit (*y* = *Ax*^*B*^, herein referred to as ‘power I’) and a vertically translated power fit (*y* = *Ax*^*B*^ + *C*, herein referred to as ‘power II’). In the determination of the power II fits, the value for *C* was fixed for each species, taken as the value of *C* when a power II fit was applied to the pooled data for all species. By restricting the number of unknown coefficients to two (*A* and *B*), this eliminates the potential for spurious results in nonlinear regression, resulting from multiple local minima in the sum of squared residuals (see [89]). Which particular type of fit best explained the data for a given variable relied primarily on the Akaike Information Criterion, applying a majority-rules consensus over the 12 species. The influence of phylogeny and body mass on the values of *A* and *B* were analysed using phylogenetic generalized least squares regressions using the ‘caper’ package in R [90], and these were taken into account when plausible (given the objective of maximizing generality). For phylogenetic analyses, a composite phylogeny of birds based on recent studies was used, and branch lengths were set as time since divergence (see S1 Appendix). For all statistical analyses, the significance level was set to *P* = 0.05.

The predictive equations concerning morphometrics, kinematics and Fourier coefficients derived using the above statistical analyses are herein collectively referred to as the biomechanically informative, regression-derived statistical model, or BIRDS Model for short. An implementation of the BIRDS Model in MATAB code is provided in the Supporting Information. The validity of the BIRDS Model was assessed by comparing its predictions against the original data, determining its internal consistency with respect to kinematic variables, and ascertaining its efficacy in extrapolation beyond the body mass range of the species studied (see S1 Appendix).

## Results

A total of 26 variables were found to vary significantly with increasing speed in birds. Not one of these concerned the mediolateral component of the GRF, *F*_*y*_. All variables that changed with increasing speed in birds were found to do so in a continuous manner. In contrast, all but one of these variables changed discontinuously with speed in humans, at the walk-run transition. Of the 52 equation parameters (26 pairs of *A* and *B*) used to describe how variables scaled with speed in birds, as detailed below, only nine of them (17.3%) showed a significant influence of phylogeny. Moreover, for these variables the *K* statistic never exceeded 1.2, indicating minor phylogenetic influence on these variables. Additionally, body mass did not show a significant influence of phylogeny, according to the *K* statistic of Blomberg et al. [91] (*K* = 0.168, *P* = 0.429).

### Morphometric relationships

**Fig 1:**
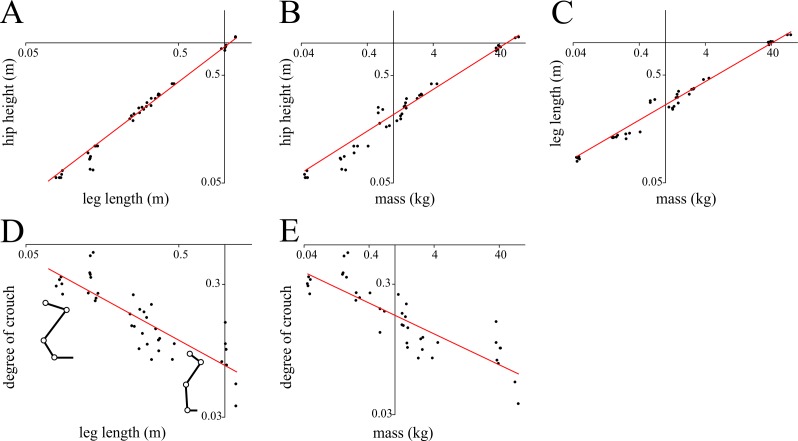
Morphometric scaling observed in the birds investigated in the current study. **(**A) Hip height *versus* leg length. (B) Hip height *versus* body mass. (C) Length *versus* body mass. (D) Degree of crouch *versus* leg length; stick figure representations show the range of crouched (e.g., quail) to erect (e.g., ostrich) postures exhibited by birds. (E) Degree of crouch *versus* body mass. All comparisons are plotted on logarithmic scales. Note that in (A)–(C), the line of best fit was determined by applying a power I fit on the untransformed variables, rather than applying a linear fit to the log-transformed values. This helped produce better results for the upper end of the body mass spectrum. Regression equations are reported in Table 2.

Hip height and leg length scaled tightly with each other and body mass in the birds studied (Fig 1A–1C; Table 2). Yet, the fitted equation for hip height *versus* leg length is apparently inappropriate for extrapolation to large body sizes: a *Tyrannosaurus* with a total leg length of 3.1 m would be predicted to have a standing hip height of 3.11 m, which is implausible. This error may stem from a bias in the underlying data (no species > 100 kg in mass) or alternatively differences in limb anatomy, such as different limb segment proportions [92].

**Table 2:** Relationships between morphometric variables in the birds studied.

Relationship	Equation	*r*^2^
hip height *versus* leg length	*h* = 0.9158·*L*^1.0794^	0.98
hip height *versus* body mass	*h* = 0.2168·*m*^0.3883^	0.99
leg length *versus* body mass	*L* = 0.2657·*m*^0.3570^	0.99
degree of crouch *versus* leg length	log_10_*DC* = -0.6306·log_10_*L* – 1.1332	0.72
degree of crouch *versus* body mass	log_10_*DC* = -0.2363·log_10_*m* – 0.7615	0.72

Note that as the objective of the current study is prediction, the power equations were fit as power equations (not as allometry equations). Consequently, 95% confidence intervals for equation coefficients cannot be generated here.

An alternative estimation of hip height is based on the fact that larger parasagittal animals stand with more erect (less crouched) postures [21,51], which is also borne out in the current study. In the birds studied here, the degree of crouch decreased predictably, and asymptotically, with increases in both leg length and body mass (Fig 1D and 1E; Table 2), despite marked differences in limb proportions between species. As leg length is known with certainty, degree of crouch may be reliably estimated from this, and subsequently standing hip height, as

h=L(1−DC). (10)

For a *Tyrannosaurus* with *L* = 3.1 m, *h* is predicted to be 2.99 m, a more plausible estimate that would necessitate a limb segment to be inclined on average 15.3° from the vertical. It needs to be emphasized that these estimations concern the height of the hip during *standing*. Standing hip height would almost certainly be different from hip height at a given instant of the stance phase of locomotion, particularly during bouncing kinematics when the leg would undergo appreciable compression [10,46,75]. As such, the above method of estimation should not be used as an explicit starting point for reconstructing limb poses throughout the stride cycle.

### Influence of speed on kinematic variables

**Fig 2:**
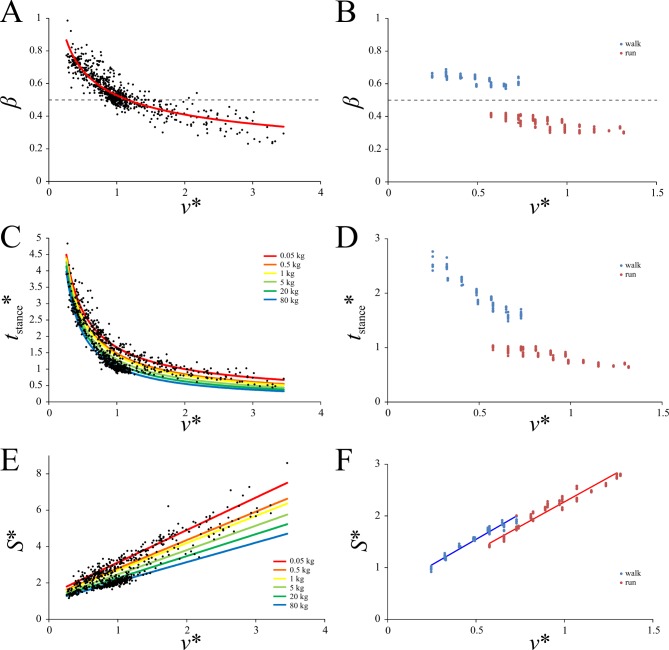
Speed scaling of kinematic variables in birds and humans. (A, B) Duty factor; horizontal dashed lines at *β* = 0.5 mark the transition from grounded to aerial locomotion. (C, D) Relative stance duration. (E, F) relative stride length. (A), (C) and (E) are for birds, (B), (D) and (F) are for humans. In (C) and (E), the relationship with speed varies with body mass, and so this has been demonstrated with several different masses. In (F), major axis fits for both walking and running have been applied; they have significantly different elevations. Regression equations are reported in Table 3.

In birds, both *β* and *t*_stance_* decreased with increasing v* in a curvilinear fashion, and in the case of *t*_stance_*, a significant influence of body mass was detected (Fig 2, Table 3). These variables also decreased with increasing v* in humans, but with a marked discontinuity at the walk-run transition (Fig 2). Relative stride length in birds increased with speed in a linear fashion, with the nature of the relationship modulated by body mass (Fig 2, Table 3). A similar linear increase was also present in humans. Although no prominent discontinuity is immediately apparent at the walk-run transition, a test of the major axis (MA) lines of best fit for the walking and running data points did reveal a significant difference in the line elevations (Fig 2F; *P* < 0.0001), as calculated using the ‘smatr’ package in R [93].

**Table 3:** Speed scaling of kinematic and kinetic variables in birds. The relationships identified between each variable and speed is of one of three forms: *y* = *Ax* + *B* (linear), *y* = *Ax*^*B*^ (power I) or *y* = *Ax*^*B*^ + *C* (power II). The relationship may also be modulated by mass, as indicated by the numbers in brackets. For each relationship, the *r*^2^ values are reported, as well as the *K* statistic of Blomberg et al. [91] and associated *P*-value for both coefficients *A* and *B*. Values in italics are statistically significant.

Variable	Fit type	*A*	*B*	*C*	*r*^2^	*K*_*A*_	*K*_*B*_	*P*_*A*_	*P*_*B*_
*β*	power I^1^	0.5278	-0.3651	n/a	0.79	0.0292	0.1438	0.834	0.439
*t*_stance_*	power I	[1]	[2]	n/a	0.93	1.0556	0.2989	*0*.*003*	0.158
S*	linear	[3]	[4]	n/a	0.92	0.0857	0.742	0.522	*0*.*015*
*F*_*x*,peak_^+^	linear^1^	0.0862	0.1341	n/a	0.23	0.0211	0.0305	0.895	0.813
*F*_*x*,peak_^–^	linear^1^	-0.1686	-0.1550	n/a	0.30	0.0142	0.0169	0.976	0.917
*F*_*z*,peak_	linear^1^	0.8530	0.8848	n/a	0.72	0.9254	0.7404	*0*.*013*	*0*.*046*
*F*_*z*,mean_	linear^1^	0.3551	0.5775	n/a	0.79	0.148	0.3032	0.379	0.26
*F*_net,peak_	linear^1^	0.7979	0.8684	n/a	0.77	0.6369	0.7426	0.097	0.055
*t*(*F*_*x*,peak_^+^)	power I^1^	0.6675	-0.1011	n/a	0.16	0.0207	0.0409	0.864	0.758
*t*(*F*_*x*,peak_^–^)	linear^1^	-0.0123	0.1794	n/a	0.01	0.4129	0.6723	0.179	0.41
*t*(*F*_*x*_ = 0)	power I^3^	[5]	-0.0705	n/a	0.34	0.0376	0.0418	0.839	0.746
*F*_*z*_(*F*_*x*_ = 0)	linear^2^	1.0326	0.5055	n/a	0.67	0.8911	0.778	*0*.*04*	0.037
*F*_*x*,MS_	linear^1^	0.0821	0.0099	n/a	0.28	0.0151	0.0174	0.962	0.856
*F*_*z*,MS_	linear^2^	0.8300	0.5230	n/a	0.58	0.315	0.845	0.232	*0*.*007*
*F*_net,MS_	linear^2^	0.8407	0.5234	n/a	0.60	0.2794	0.5748	0.283	*0*.*036*
*Xa*_2_	power II^1^	-0.0082	3.0997	-0.165	0.28	0.0188	0.2893	0.939	0.358
*Xa*_3_	linear^1^	-0.0593	-0.0289	n/a	0.25	0.0187	0.0202	0.921	0.879
*Xa*_4_	linear^1^	-0.0044	-0.0275	n/a	-0.02	1.0881	0.0224	*0*.*002*	0.918
*Xa*_5_	linear^1^	-0.0008	-0.0327	n/a	0.0001	0.2117	0.0301	0.318	0.847
*Za*_1_	linear^1^	0.5897	0.8725	n/a	0.74	0.2393	0.348	0.234	0.207
*Za*_2_	power II^1^	0.8899	0.4370	-0.532	0.17	0.3202	0.2308	0.211	0.501
*Za*_3_	linear^2^	-0.2162	0.3127	n/a	-0.08	0.8603	0.3537	*0*.*026*	0.159
*Za*_4_	linear^1^	-0.0693	0.1401	n/a	0.64	0.5474	0.145	0.136	0.493
*Za*_5_	linear^1^	0.0033	-0.0456	n/a	0.001	0.1175	0.4454	0.461	0.083
*Za*_6_	linear^1^	-0.0264	0.0225	n/a	0.16	0.0938	0.3557	0.588	0.208
NVD*	power I^1^	0.0560	0.4764	n/a	0.28	0.0991	0.0298	0.558	0.835

[1] = -0.1841·log_10_*m* + 1.4265

[2] = -0.0725·log_10_*m* – 0.8266

[3] = -0.2226·log_10_*m* + 1.4957

[4] = -0.1058·log_10_*m* + 1.2004

[5] = 0.0391·log_10_*m* + 0.3939

^1^ = model is an all-species fit.

^2^ = *A* and *B* are means across species.

^3^ = *B* is a mean across species.

### Influence of speed on the GRF

**Fig 3:**
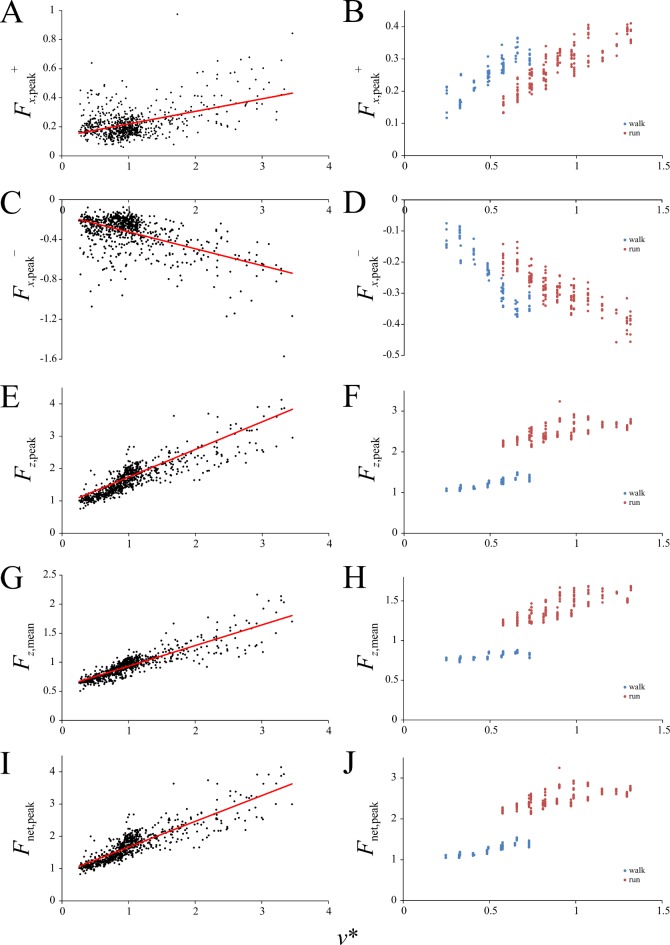
Speed scaling of peak force variables in birds and humans. Forces are normalized to body weight. (A, B) Positive peak *F*_*x*_. (C, D) Negative peak *F*_*x*_. (E, F) Peak *F*_*z*_. (G, H) Mean *F*_*z*_. (I, J) Peak net force. (A), (C), (E), (G) and (I) are for birds, (B), (D), (F), (H) and (J) are for humans. Regression equations for birds are reported in Table 3.

**Fig 4:**
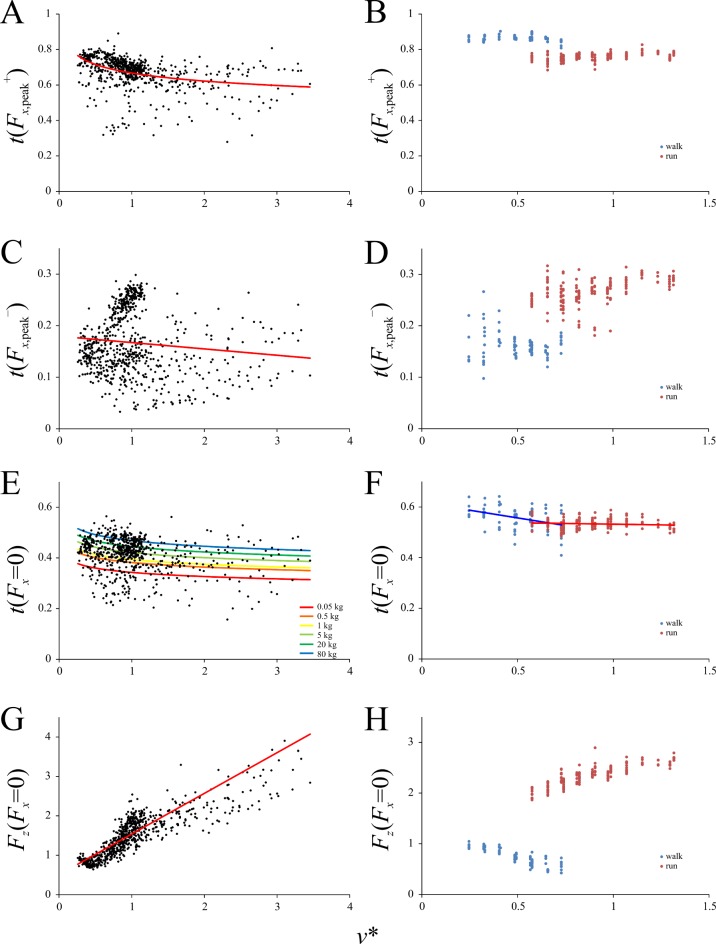
Speed scaling of temporal variables concerning the GRF in birds and humans. Times are normalized to the duration of stance. (A, B) Time of positive peak *F*_*x*_. (C, D) Time of negative peak *F*_*x*_. (E, F) Time at which *F*_*x*_ is zero. (G, H) The magnitude of *F*_*z*_ when *F*_*x*_ is zero. (A), (C), (E) and (G) are for birds, (B), (D), (F) and (H) are for humans. In (E), the relationship with speed varies with body mass, and so this has been demonstrated with several different masses. In (F), major axis fits for both walking and running have been applied; they have the same elevation at the walk-run transition. Regression equations for birds are reported in Table 3.

**Fig 5:**
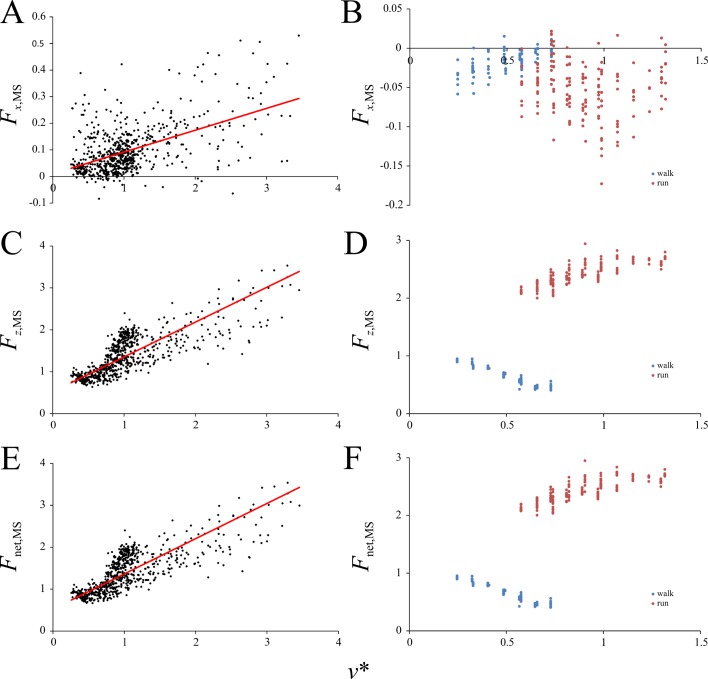
Speed scaling of the GRF at mid-stance. (A, B) The anteroposterior component at mid-stance. (C, D) The vertical component at mid-stance. (E, F) The net GRF at mid-stance. (A), (C) and (E) are for birds, (B), (D) and (F) are for humans. Regression equations are reported in Table 3.

**Fig 6:**
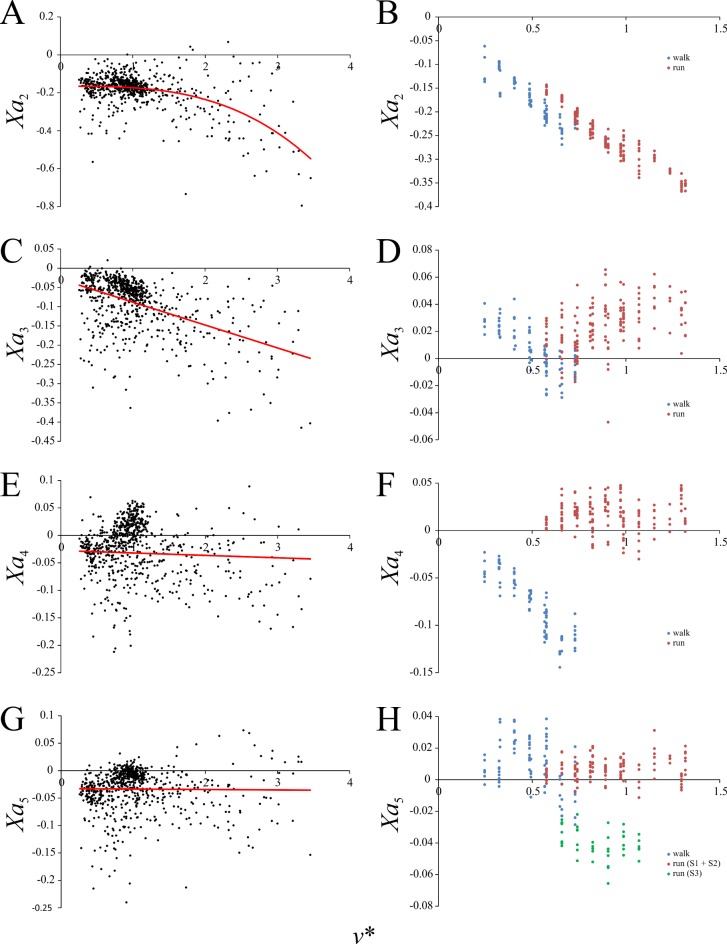
Speed scaling of Fourier coefficients describing the anteroposterior component of the GRF. (A, B) *Xa*_2_. (C, D) *Xa*_3_. (E, F) *Xa*_4_. (G, H) *Xa*_5_. (A), (C), (E) and (G) are for birds, (B), (D), (F) and (H) are for humans. Regression equations are reported in Table 3.

**Fig 7:**
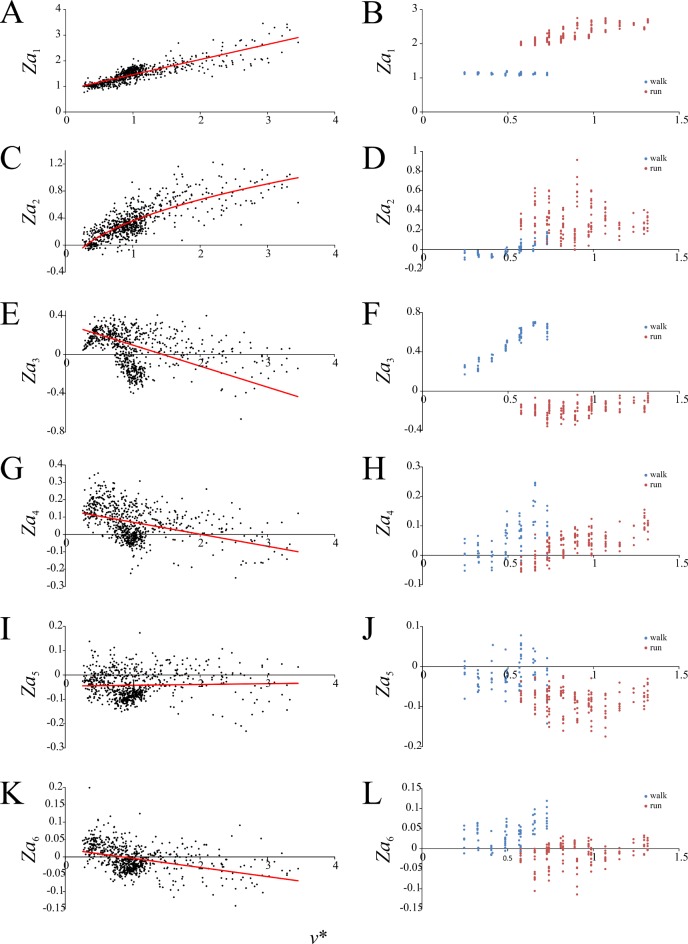
Speed scaling of Fourier coefficients describing the vertical component of the GRF. (A, B) *Za*_1_. (C, D) *Za*_2_. (E, F) *Za*_3_. (G, H) *Za*_4_. (I, J) *Za*_5_. (K, L) *Za*_6_. (A), (C), (E), (G), (I) and (K) are for birds, (B), (D), (F), (H), (J) and (L) are for humans. Regression equations are reported in Table 3.

The effect of speed on various features of the GRF profile in birds is reported in Table 3 and Figs 3–7; the results for humans are also reported in Figs 3–7. Both the positive (*F*_*x*,peak_^+^) and negative (*F*_*x*,peak_^–^) peak magnitudes of the anteroposterior component of the GRF increased linearly with increasing v* (Fig 3A–3D), as did the peak and mean magnitudes of vertical component over the stance (*F*_*z*,peak_ and *F*_*z*,mean_, respectively; Fig 3E–3H) and the net GRF (*F*_net,peak_; Fig 3I and 3J). For each variable a similar pattern of increase was also observed in the humans, but a distinct discontinuity at the walk-run transition was ubiquitous.

The timing of the positive and negative peak magnitudes of the anteroposterior component of the GRF, *t*(*F*_*x*,peak_^+^) and *t*(*F*_*x*,peak_^–^) respectively, both decreased slightly with increasing v*, that is, they occured earlier in the stance as speed increased (Fig 4A–4D), although there was a great amount of scatter. Humans showed a distinct discontinuity at the walk-run transition in both of these features. Importantly, *t*(*F*_*x*,peak_^+^) for humans tended to occur later in the stance compared to birds (0.7–0.9 *versus* 0.45–0.8; *t*-test: *t* = 20.046, *P* <0.0001), and in a running gait, *t*(*F*_*x*,peak_^–^) for humans tended to be higher than for birds (0.2–0.3 *versus* 0.05–0.25; *t*-test: *t* = 14.196, *P* < 0.0001). That is, at faster speeds, the instances of *F*_*x*,peak_^+^ and *F*_*x*,peak_^−^tended to occur earlier on in the stance phase in birds than humans.

The instant at which the anteroposterior component of the GRF is zero, *t*(*F*_*x*_ = 0), is the instant at which the GRF is vertical in the sagittal plane. The value of *t*(*F*_*x*_ = 0) gradually decreased with increasing v* in birds in a gently curvilinear fashion, and body mass modulated this relationship slightly (Fig 4E). In humans *t*(*F*_*x*_ = 0) also decreased gradually with increasing v*. Although the MA lines of best fit for walking and running in humans had significantly different slopes (*P* < 0.0001, calculated using the ‘smatr’ package), the two had similar elevations at the walk-run transition (Fig 4F). Thus, the transition from walking to running was not abruptly discontinuous as observed for the other parameters investigated here. Importantly, *t*(*F*_*x*_ = 0) was almost always less than 0.5 in birds, particularly in smaller species (i.e., event occurred in the first half of stance), and was almost always greater than 0.5 in humans (i.e., event occurred in the second half of stance).

The magnitude of the vertical component of the GRF at this instance in the stance phase, *F*_*z*_(*F*_*x*_ = 0), increased linearly with increasing v* in birds (Fig 4G). In humans, *F*_*z*_(*F*_*x*_ = 0) *decreased* with increasing v* in walking, abruptly and substantially increased at the walk-run transition, and then increased with increasing v* at running speeds (Fig 4H). The reason for the decrease with v* during walking is seen in the fact that the force-time profile of Fz became progressively more double-peaked at faster walking speeds (see below), with the trough between the two peaks moving closer to the abscissa. As the trough occurs at around a similar time to *t*(*F*_*x*_ = 0), the magnitude of *F*_*z*_(*F*_*x*_ = 0) consequently decreased with increasing speed.

At mid-stance (i.e., the point when *t* = 0.5*t*_stance_), the magnitudes of the anteroposterior (*F*_*x*,MS_) and vertical (*F*_*z*,MS_) components of the GRF, as well as the net magnitude (*F*_net,MS_), all increased linearly with increasing v* in birds (Fig 5). In humans, *F*_*x*,MS_ increased with increasing v* in walking and decreased with increasing v* in running, the two separated by a discontinuity. As a consequence of the difference in *t*(*F*_*x*_ = 0) between humans and birds, and the fact that *t*(*F*_*x*_ = 0) is relatively close to 0.5 in humans, *F*_*z*,MS_ and *F*_net,MS_ in humans followed a similar pattern as observed for *F*_*z*_(*F*_*x*_ = 0). Furthermore, that *t*(*F*_*x*_ = 0) was almost always less than 0.5 in birds means that *F*_*x*,MS_ was almost always positive (Fig 5A), whereas in humans it was almost always negative (Fig 5B).

Power analysis of the Fourier coefficients for all three components of the GRF revealed that the relative magnitude of the power of the cosine and sine terms (= *b*_*n*_^2^/*a*_*n*_^2^) was always less than 0.7%. That is, the relative contribution of the cosine terms to the explanatory power of the Fourier series is negligible; the force-time profiles could be sufficiently described using only sine (*a*) terms. Additionally, power analysis revealed that 9.34 ± 4.18 (mean ± s.d.) fundamental frequencies contain 99% or more of the signal’s power in terms of *F*_*x*_, 11.58 ± 7.13 fundamental frequencies contain 99% or more of the signal’s power in terms of *F*_*y*_, and that 3.20 ± 1.04 fundamental frequencies contain 99% or more of the signal’s power in terms of *F*_*z*_. These results indicate that, by and large, 99% (or more) of the force-time profile was able be explained by the first ten or fewer fundamental frequencies.

Four sine-based Fourier coefficients for describing the force-time profile of *F*_*x*_ were found to be significantly different from zero and varied significantly with increasing v*. These relate to the frequencies of 2–5 Hz, and are herein referred to as *Xa*_2_, *Xa*_3_, *Xa*_4_ and *Xa*_5_. Hence, the *F*_*x*_ force-time profile can be described thus:

$$F_{x}(t)=\sum_{n=2}^{5}Xa_{n}\sin(\pi nt),$$ (11)

where *F*_*x*_ and *t* are normalized to body weight and stance duration, respectively. In birds the value of *Xa*_2_ decreased with increasing v* in a curvilinear fashion, whereas it decreased linearly in humans, with a small discontinuity occurring at the walk-run transition (Fig 6A and 6B). The value of *Xa*_3_ and *Xa*_4_ decreased continuously with increasing v* in birds, although only gradually in the case of *Xa*_4_. The values for both coefficients tended to be negative for birds, whereas in humans *Xa*_3_ and *Xa*_4_ at running speeds tended to be positive (Fig 6C–6F). Moreover, marked discontinuities occurred at the walk-run transition for both coefficients in humans. *Xa*_5_ in birds increased very slightly with increasing v*, but remained largely negative, whereas in humans it was largely positive across walking and running (Fig 6G and 6H). Humans again showed a discontinuity with speed in this variable at the walk-run transition, although intriguingly in one subject (subject S3) the discontinuity was more pronounced than in the other two (Fig 6H).

Six sine-based Fourier coefficients for describing the force-time profile of *F*_*z*_ were found to be significantly different from zero and varied significantly with increasing v*. These relate to the frequencies of 1–6 Hz, and are herein referred to as *Za*_1_, *Za*_2_, *Za*_3_, *Za*_4_, *Za*_5_ and *Za*_6_. Hence, the *F*_*z*_ force-time profile can be described thus:

$$F_{z}(t)=\sum_{n=1}^{6}Za_{n}\sin(\pi nt),$$ (12)

where *F*_*z*_ and *t* are normalized to body weight and stance duration, respectively. In birds the value of *Za*_1_ and *Za*_3_–*Za*_6_ changed with increasing v* in a linear fashion, whereas the value of *Za*_2_ changed in a slight curvilinear fashion with respect to v* (Fig 7). In humans each of these variables changed with increasing *v* in a discontinuous fashion, with marked changes in values occurring at the walk-run transition. The pattern of change was not always congruent between bird and humans; for instance, *Za*_4_ decreased with increasing v* in birds, but within both walking and running gaits in humans it increased with increasing v* (Fig 7G and 7H).

**Fig 8:**
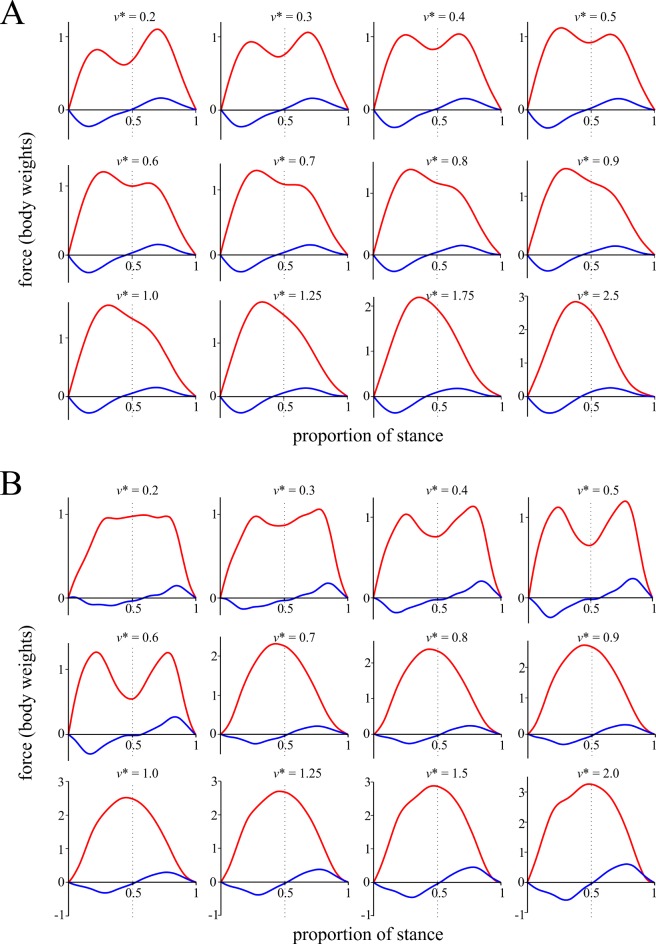
Change in the shape of the GRF force-time profiles with increasing speed. (A) Pattern of change observed in birds. (B) Pattern of change observed in humans. Red profile is vertical component, blue profile is anteroposterior component. Among other things, note how the profiles for birds show marked temporal asymmetry compared to those of humans, with more force being applied in the first half of stance. For humans, force-time profiles were calculated from least squares linear fits applied to the respective data, with the exception of *Za*_2_ for walking, which was better fit by a power II model. The predicted curves for both components are based on the first ten sine coefficients (i.e., *Xa*_1_–*Xa*_10_, *Za*_1_–*Za*_10_), all of which either showed significant variation with speed or significantly non-zero values (or both).

Using the relationships identified between each of the Fourier coefficients and v* in birds, the shape of the *F*_*x*_ and *F*_*z*_ force-time profiles can be calculated with the equations for *F*_*x*_(*t*) and *F*_*z*_(*t*) respectively (Fig 8A). Not surprisingly, the shapes of the force-time profiles change continuously with increasing v*, as all of the Fourier coefficients also change continuously with v*. The most important changes occur to the *F*_*z*_ profile. At slow speeds the *F*_*z*_ profile was double-peaked; at very slow speeds the second peak was larger than the first, but as v* increased, the first peak became more prominent and the second peak atrophied. This continued with faster speeds until the second peak vanished completely, leaving an asymmetric profile. For comparison, the *F*_*x*_ and *F*_*z*_ force-time profiles were also calculated for humans (Fig 8B), and they showed a marked change in shape at the walk-run transition, at relative speeds of 0.6–0.7. At slow walking the *F*_*z*_ profile was double-peaked, and became progressively more double-peaked as walking speed increased. At the transition to running, the double peaks were replaced by a single, nearly symmetrical peak which was retained as v* increased further, although the presence of an initial impact transient became more prominent at higher speeds. These patterns for humans are entirely consistent with those reported by previous studies [72,94–103].

### Influence of speed on mechanical energy fluctuations

**Fig 9:**
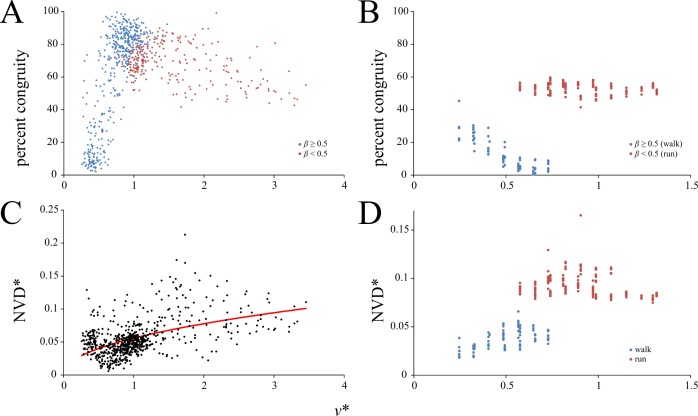
Speed scaling of mechanical energy fluctuations. (A, B) percent congruity, distinguished by duty factor. (C, D) normalized net vertical displacement of the COM. (A) and (C) are for birds, (B) and (D) are for humans. Regression equation for (C) is reported in Table 3.

Percent congruity of KE and PE, as calculated from simulations of whole-stride GRFs, showed an intriguing pattern in birds (Fig 9A). At the slowest speeds recorded, it could take on a range of values up to 70%, but in general was considerably less than that. It increased continuously from low values toward high values as v* increased, reaching a maximum (almost 100% in some trials) at relative speeds of about v* ≈ 1. However, beyond v* ≈ 1, it decreased again with further increase in v*, albeit at a lessened rate. The inflection at v* ≈ 1 may therefore be seen, at face value, to be a discontinuity with respect to speed. A similar pattern has also been reported in birds for other, analogous measures of mechanical energy fluctuations, namely, phase shift and pendular energy recovery [28,36,37,46]. The pattern of percent congruity change with increasing v* in humans was markedly different to that seen in birds (Fig 9B). In walking it actually decreased with increasing speed before suddenly increasing again at the walk-run transition; whereupon it remained at a fairly constant value of 50–60%.

The normalized net vertical displacement (NVD*) for birds had a considerable amount of scatter, but nevertheless increased with increasing v*, being best fit by a power I relationship (Fig 9C). The variance with respect to v* was more tightly constrained in humans, and NVD* also increased with increasing v*, although again a marked discontinuity occurred at the walk-run transition (Fig 9D). It also appears that at the fastest speeds tested for humans, NVD may decrease somewhat. Although the analysis of GRFs in this study explicitly assumed equal force distribution by both feet during locomotion, this may not always be the case, for small birds at least [45]. If some of the species examined in the present study used ‘mixed gaits’ at certain speeds [45], this may exaggerate the variation in calculated values for percent congruity and NVD* reported here.

### Other important features

**Fig 10:**
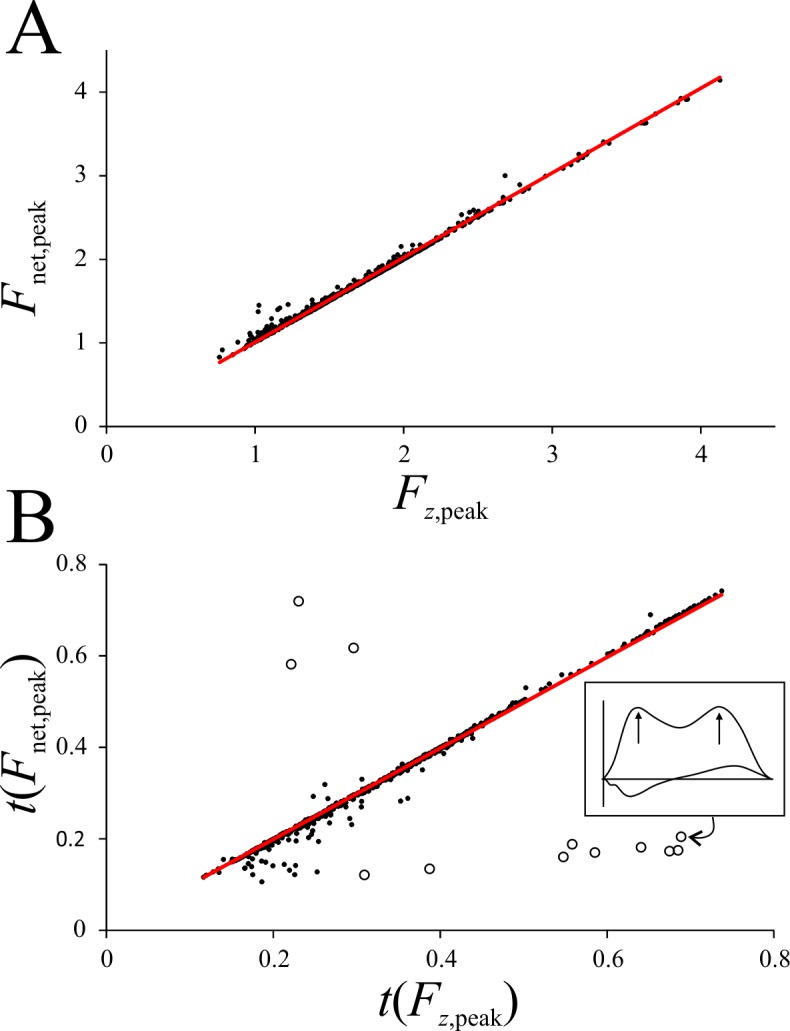
The dominance of the vertical component of the GRF. (A) Peak net force scales very strongly with peak vertical force; major axis line has a slope almost exactly equal to unity. (B) The instance of peak net force is very strongly coincident with the instance of peak vertical force; major axis line has a slope almost exactly equal to unity. In B, the outliers (hollow circles) are slow speed trials in which the two peaks were of almost the same magnitude, such that the peak net force can occur at a very different time to when the vertical component is at a maximum (inset illustrates one such trial).

Unsurprisingly, the vertical force was by far the dominant component of the GRF in birds, as seen by the fact that *F*_*z*,peak_ scaled very strongly linearly with *F*_net,peak_, very close to the line of parity (Fig 10A; MA regression forced through the origin has a slope of 1.0118, *r*^2^ = 0.995). Additionally, across the 701 trials analyzed, the peak magnitude of *F*_*x*_ and *F*_*y*_ was on average 21.15 ± 11.46% and 9.83 ± 6.97% of *F*_*z*,peak_, respectively. The instance of *F*_*z*,peak_, *t*(*F*_*z*,peak_) was very strongly associated with the instance of *F*_net,peak_, *t*(*F*_net,peak_) (Fig 10B; MA regression forced through the origin has a slope of 0.99518, *r*^2^ = 0.993).

**Fig 11:**
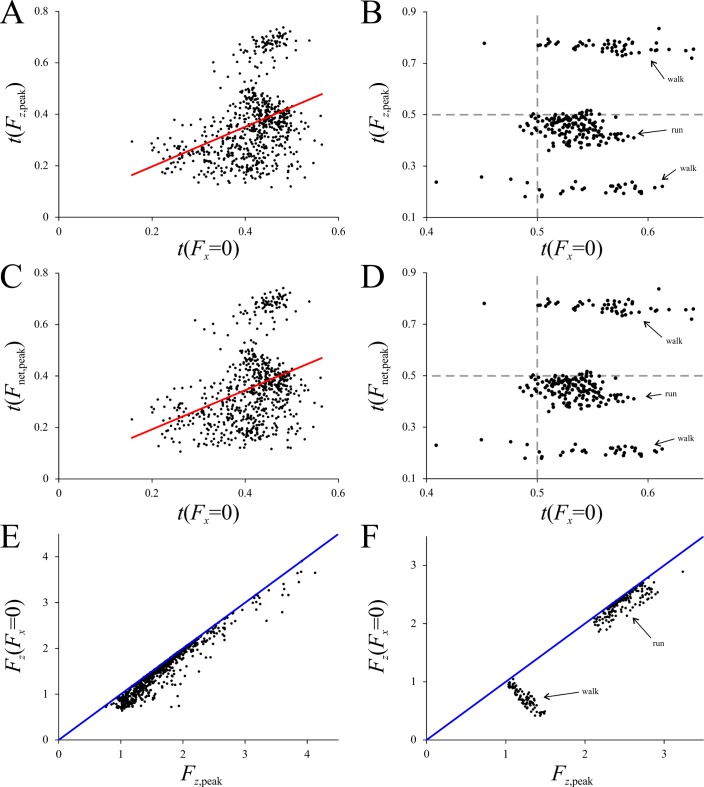
The association, or lack thereof, between *t*(*F*_z,peak_) or *t*(*F*_net,peak_) and *t*(*F*_*x*_ = 0). (A, B) *t*(*F*_*z*,peak_) *versus t*(*F*_*x*_ = 0). (C, D) *t*(*F*_net,peak_) *versus t*(*F*_*x*_ = 0). (E, F) *F*_*z*_(*F*_*x*_ = 0) compared to *F*_*z*,peak_ for each trial; the blue lines are lines of parity. (A), (C) and (E) are for birds, (B), (D) and (F) are for humans. In (A), the regression line has a slope of 0.7718 and an *r*^2^ of 0.1289; in (C), the regression line has a slope of 0.7627 and an *r*^2^ of 0.1194. In human walking, peak vertical or net force either occurs early or late in the stance; in running, they occur largely before mid-stance (*t* < 0.5), yet *t*(*F*_*x*_ = 0) largely occurs after mid-stance (*t* > 0.5).

In birds, *t*(*F*_*z*,peak_) and *t*(*F*_net,peak_) appeared to be weakly coincident with *t*(*F*_*x*_ = 0) (Fig 11A and 11C). This was not the case in humans, where no correlation existed at all (Fig 11B and 11D). To formally test for a correlation between *t*(*F*_*z*,peak_) or *t*(*F*_net,peak_) with *t*(*F*_*x*_ = 0) in birds, this would usually involve computation of a linear regression and an associated significance value, based on a parametric statistical test. This could not be done in the current situation, however, for the data were found to fail two assumptions of standard parametric tests of the slope, as determined in PAST, namely, non-normal distribution of errors and heteroscedasticity (using the Breusch-Pagan test). Instead, a permutation test was used [104], implemented in a custom MATLAB script. For both *t*(*F*_*z*,peak_) *versus t*(*F*_*x*_ = 0) and *t*(*F*_net,peak_) *versus t*(*F*_*x*_ = 0), the least-squares slope was determined; the values of *t*(*F*_*z*,peak_) or *t*(*F*_net,peak_) were then randomly rearranged with respect to *t*(*F*_*x*_ = 0) and the slope recomputed. A total of 100,000 replicates were conducted for both data tests. The significance (*P*-value) of the slope is the proportion of replicates for which the calculated slope is equal to or more extreme than the actual slope of the data. In both cases, this revealed the slope to be significant (*P* < 0.0001). Despite this, the *r*^2^ values in both cases were rather poor, with less than 13% of the variation explained by *t*(*F*_*x*_ = 0): there was only a weak association between *t*(*F*_*z*,peak_) or *t*(*F*_net,peak_) with *t*(*F*_*x*_ = 0). Therefore, in both birds and humans, the instant of peak vertical force or peak net GRF was not necessarily associated with the instant at which the GRF vector is vertical in the sagittal plane. Consequently, whilst *F*_*z*_(*F*_*x*_ = 0) could be equal or very close to *F*_*z*,peak_ (or *F*_net,peak_), it was almost always less than that, and often significantly so (Fig 11E and 11F).

**Fig 12:**
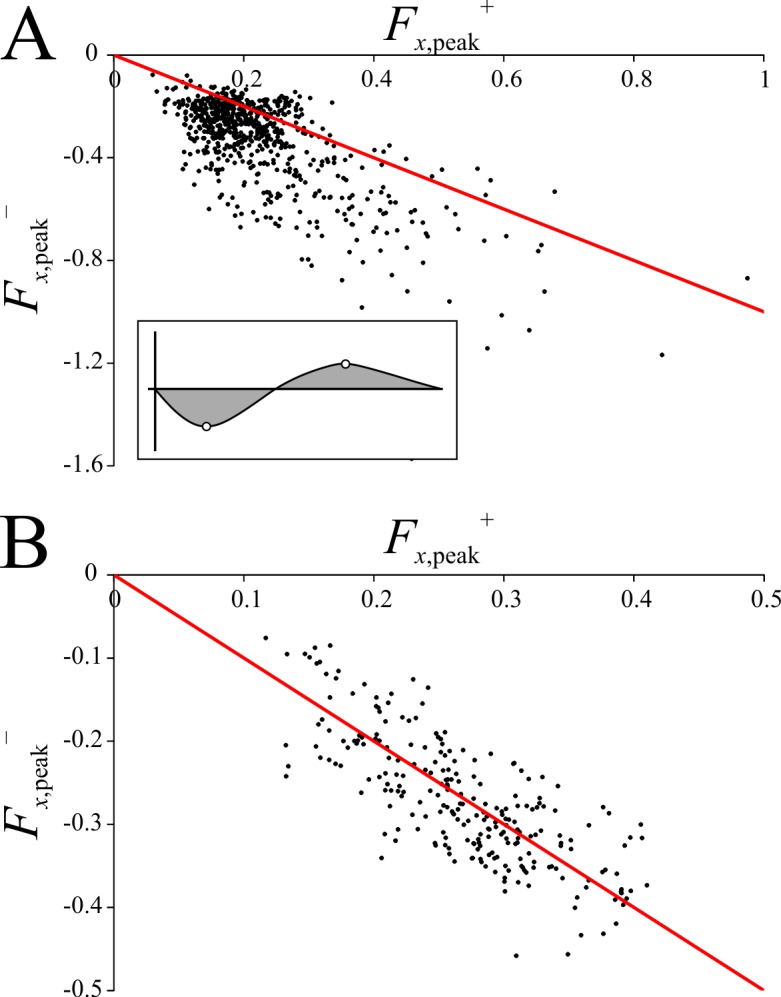
A comparison of peak positive and negative magnitudes of the anteroposterior component of the GRF. (A) *F*_*x*,peak_^−^*versus F*_*x*,peak_^+^ in birds; the vast majority of data points fall below the line of negative parity. (B) *F*_*x*,peak_^−^*versus F*_*x*,peak_^+^ in humans; the data points fall neatly on the line of negative parity. In (A), the inset shows how differing durations of deceleration and acceleration phases require different peak magnitudes, in order for impulses (shaded area) to remain balanced.

A comparison of *F*_*x*,peak_^−^against *F*_*x*,peak_^+^ in birds revealed that the data points almost always fell below the line of (negative) parity (Fig 12A); that is, |*F*_*x*,peak_^–^| was almost always greater than *F*_*x*,peak_^+^ (paired *t*-test: *t* = 21.727, *P* < 0.0001). This is a consequence of the fact that *t*(*F*_*x*_ = 0) was almost always substantially less than 0.5 in birds (see above). In order for the deceleration (negative *F*_*x*_) phase of the stance to be shorter in duration than the acceleration (positive *F*_*x*_) phase, and yet impulses remain approximately balanced, this requires the deceleration component to reach a greater peak value (Fig 12A inset). In contrast to the birds, |*F*_*x*,peak_^–^| in humans was not significantly different from *F*_*x*,peak_^+^ (paired *t*-test: *t* = 1.125, *P* = 0.262); a graphical comparison of *F*_*x*,peak_^−^against *F*_*x*,peak_^+^ shows the data points to neatly fall along the line of (negative) parity (Fig 12B).

### BIRDS model validation tests

The full results of model validation are detailed in the Supporting Information (S1 Appendix), and they will only be summarized here. Overall, the BIRDS Model performed well, explaining five-sixths of the variation in the observed vertical component of the GRF and over two-thirds of the variation in the observed anteroposterior component. It also predicted individual aspects of the GRF profiles with good accuracy, in some instances out-performing the predictive equations derived individually and specifically for each variable. In terms of kinematic predictions, the model was shown to have considerable internal consistency. Extrapolation tests showed that the model’s predictions contained only a small amount of error, about 3% or less, suggesting that it is capable of making extrapolations within the margin of error of other biomechanical parameters that would be estimated for an extinct species, such as body mass or COM location [34,82,84,105].

**Fig 13:**
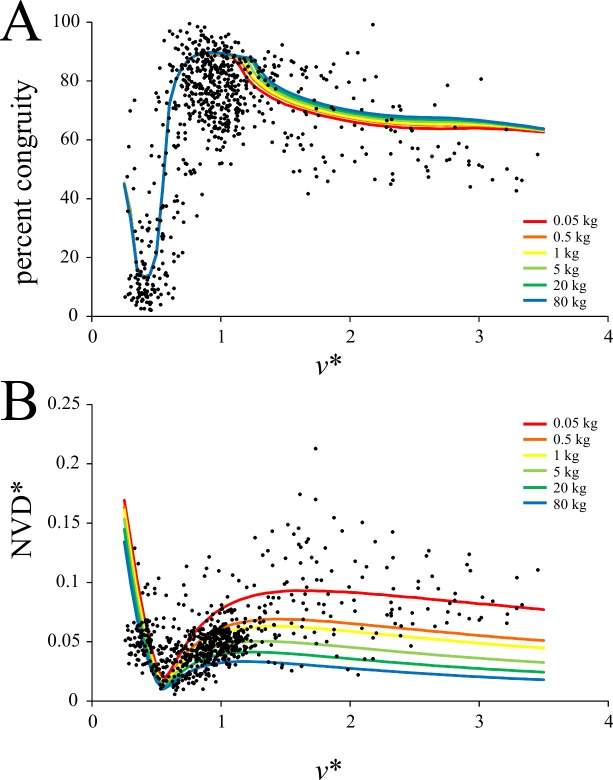
Comparison of predictions of the BIRDS model against the observed data for mechanical energy fluctuations. (A) Percent congruity; *r*^2^ of predictions is 0.509, root mean square error (RMSE) is 14.895. (B) Relative net vertical displacement of the COM; *r*^2^ of predictions is -3.569, RMSE is 0.0339. For both variables, the model predicted an influence of body mass, although this is not particularly pronounced for the prediction of percent congruity.

One other important result concerns the BIRDS Model’s predictions of mechanical energy fluctuations. The predictions again were relatively good, replicating the general pattern of change well (Fig 13). Regarding its predictions of change in percent congruity with increasing v*, two features are of note (Fig 13A). Firstly, the apparent inflection in the data at v* ≈ 1 was predicted to be a gradual change, rather than an abrupt discontinuity as seen with most variables measured in humans. Secondly, the model predicted that at very slow speeds, percent congruity increased, producing a U-shape to the initial part of the plot. A U-shape has also been reported at slow speeds for phase shift and pendular energy recovery by other studies [28,36,46], further supporting the model’s validity.

## Discussion

Through investigating twelve species of ground-dwelling bird, spanning a 1,780-fold range in body mass, this study sought to identify how speed and size modulates some basic parameters of avian terrestrial locomotion, relating to kinematics and the ground reaction force (GRF). Based on the relationships between speed or size and these parameters, equations were developed that may be used to help predict locomotion in extinct theropods. In addition, the comparison of locomotor biomechanics in birds to that for humans has further clarified the similarities and differences between the two groups, building on the findings of previous studies (c.f. [21]).

### Birds *versus* humans

**Fig 14:**
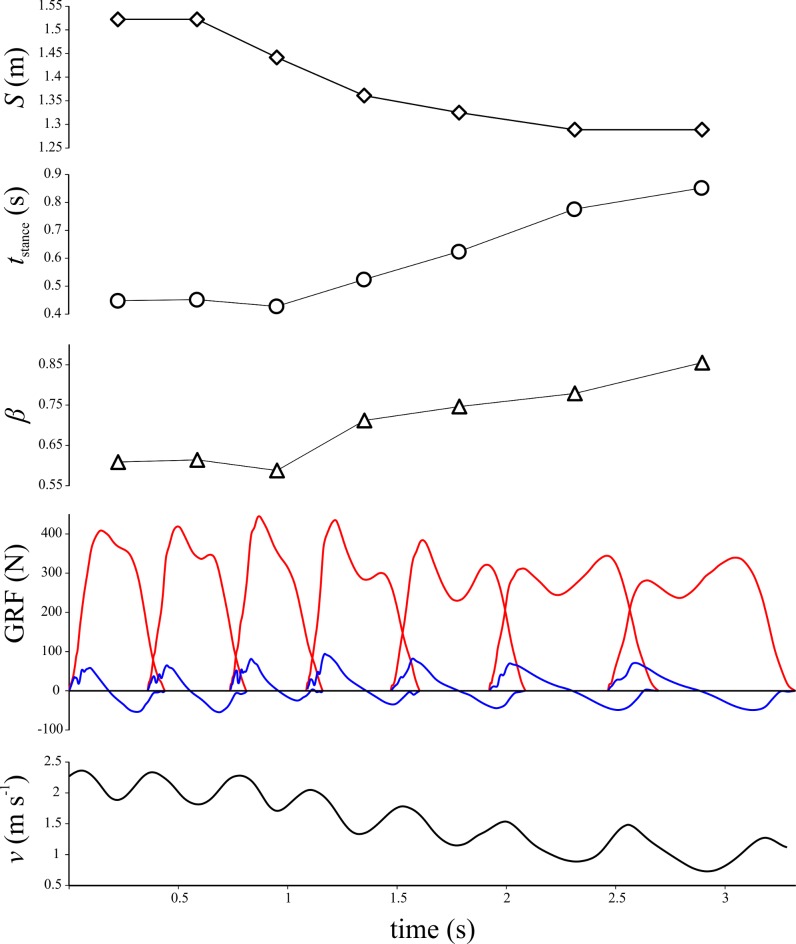
A trial of seven footfalls in which the subject (emu) underwent a gradual deceleration. Stride length, stance duration, duty factor and the nature of the GRF (blue profile = *F*_*x*_, red profile = *F*_*z*_) change continuously with continuous decrease in speed. Note that speed is shown as the instantaneous speed of the back marker.

Every variable that was found to vary with speed in birds did so continuously, whereas humans typically showed an abrupt and pronounced change, coinciding with the walk-run transition. This parallels the findings of previous studies, which have demonstrated that many kinematic parameters that change abruptly with speed in humans do so in a continuous fashion in birds [21,22,24,28,32,36–38,40,42,43,106,107]. In addition to applying across speeds, the continuous patterns of birds also appear to hold within individual trials during acceleration or deceleration; Fig 14 illustrates one such example, with an emu undergoing gradual deceleration from *v* ≈ 2.2 m s^-1^ to *v* ≈ 1.0 m s^-1^.

**Fig 15:**
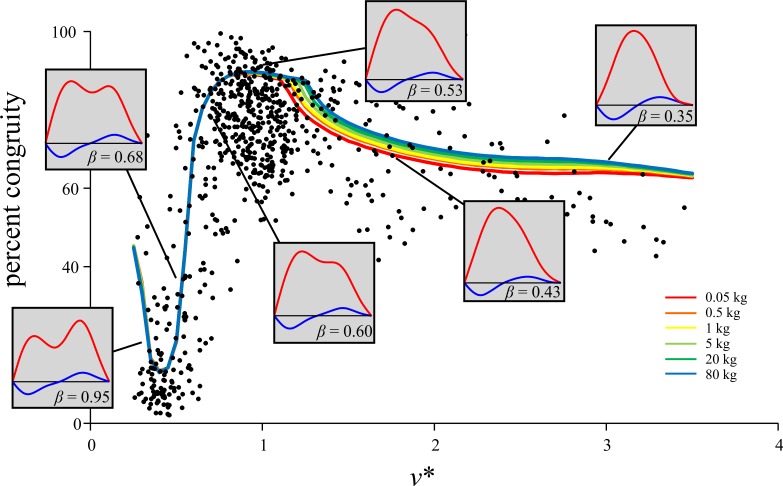
Changes in the GRF, duty factor and mechanical energy fluctuations with increasing speed. The force-time profile shape and duty factor for several different speeds have been diagrammatically mapped onto the comparison between percent congruity and speed. The predictions of the BIRDS Model for various body masses are also shown. Note how the shapes of the force-time profiles change far less beyond v* ≈ 1, yet duty factor continues to decrease.

The results for percent congruity in bird locomotion appeared to show a discontinuity with respect to speed, occurring at v* ≈ 1. However, simulations with the BIRDS Model showed that a gradual change occurred here (Fig 13A). This suggests that the apparent discontinuity observed can result from the summation and interaction of multiple continuous changes (in force-time profile shape, duty factor and *t*_stance_) occurring together (Fig 15). Furthermore, percent congruity increased continuously from low values at slow speeds (where vaulting-like mechanics dominate) up to high values at v* ≈ 1 (where bouncing-like mechanics dominate). Duty factor typically remained above 0.5 across this speed range (Figs 2A and 9A), and hence the transition from ‘walking’ to ‘grounded running’ in birds occurred within this range as well. Indeed, much of the transition from ‘walking’ through to ‘aerial running’ took place over the speed range up to v* ≈ 1. Therefore, the transition from ‘walking’ to ‘grounded running’ occurred gradually, without any abrupt change in the nature of mechanical energy fluctuations. Only once ‘aerial running’ had been attained, and speed increased further, did the nature of mechanical energy fluctuations change, and here its change with respect to speed was still continuous. The cause for the gradual decrease in percent congruity with further increases in speed beyond v* ≈ 1 may relate to the fact that above this speed, the GRF force-time profile changed much less (the *F*_*z*_ force-time profile was already single-peaked by the time v* ≈ 1), but duty factor continued to decrease with increasing speed (Fig 15).

It is worth briefly noting that percent congruity in this study was calculated based on simulated whole-stride GRF patterns, which were generated under the assumption of symmetrical force distribution between right and left limbs. However, it has been recently shown that this may not always be the case [45]. If some or all of the birds investigated in the present study did use ‘mixed gaits’ at certain speeds, this could influence estimations of mechanical energy fluctuations, perhaps explaining some of the variation in calculated percent congruity for relative speeds of 0.5–1 (Fig 9A). One outstanding issue is that all of the species studied by Andrada et al. [45] and noted as exhibiting mixed gaits were less than 600 g in body mass. Whether the phenomenon of mixed gaits is more widespread in birds, or just restricted to small-bodied species, therefore remains unknown. An additional potential source of variation in calculated values of percent congruity could be the use of head-bobbing by some or all of the birds studied [108], although this was not quantified in the present study.

In terms of kinematics, forces and energy fluctuations of the COM, this and previous studies have ubiquitously demonstrated that ground-dwelling birds exhibit a smooth transition from ‘walking’ to ‘aerial running’: they have a highly continuous locomotor repertoire. It is therefore not possible to distinguish discrete gaits at intermediate speeds, as can be done for humans and many other animals [109]. Hence, human-like walking and human-like running may be seen as end-points of the spectrum of locomotor behaviour observed in avian terrestrial locomotion.

Previously, Gatesy and Biewener [21] and Gatesy [22] identified a potential gait transition in the bird species they studied. This was on the basis that for relative stride frequency, relative step length and limb retraction angle, the *rate of change* with respect to speed was determined to change significantly at the reported transition. As noted by Rubenson et al. [36], however, the way in which these parameters change with speed could alternatively be, and perhaps better, modelled by curvilinear relationships, rather than linear ones. In this case, no distinct transition would exist. Regardless of this discrepancy, the overwhelming majority of kinematic and kinetic parameters measured in birds to date show continuous change with increasing speed.

In light of the above considerations, it is intriguing that two studies have observed pronounced discontinuities in how energy expenditure (metabolic cost of transport) change with increasing speed in large birds (emus and ostriches: [36,110]). These discontinuities were found to occur at v* ≈ 0.4–0.5, which is well within the region of continuous increase in percent congruity as calculated here. However, no such discontinuity was observed in a much smaller species (rock ptarmigan: [38]). The factor or factors responsible for these discontinuities, and whether this only occurs in large birds, remain to be determined.

One further point of interest is that whilst this study investigated only adult humans, finding many marked differences from birds, young children (< 5 years of age) may exhibit more bird-like locomotor patterns. For instance, duty factor or the shape of the force-time profile of the GRF may vary with speed in a more continuous fashion compared to adults [111]. It remains to be discerned if this phenomenon is related to smaller body size at young age in humans, or instead reflects the ontogenetic maturation of neuromuscular control of movement, but is worthy of future investigation.

### Predicting terrestrial locomotor biomechanics in theropods

The BIRDS Model developed here can be used to predict several fundamental variables involved in striding bipedal locomotion: duty factor, stance duration, stride length and the nature of the GRF in the sagittal plane throughout the stance. In turn, the nature of mechanical energy fluctuations may be ascertained. All the model requires for these predictions are two inputs, mass and speed, although if total leg length is also known this can refine the accuracy of the predictions. These predictions have been shown to perform well over the range of masses, postures and speeds studied. On average, the model can explain 79–93% of the observed variation in kinematics and 69–83% of the observed variation in GRFs. The model has also been shown to perform well in extrapolation to both higher and lower body masses. This instills confidence that it can, to some degree, be used to estimate important biomechanical parameters involved in terrestrial locomotion for extinct theropods, both avian (e.g., moas, dromornithids, phorusrhacids) and non-avian (e.g., *Tyrannosaurus*, *Allosaurus*, *Australovenator*, *Velociraptor*).

Returning to the initial question posed in the Introduction, what would an eight tonne *Tyrannosaurus* have looked like moving at 5 m s^-1^? In its current form, the BIRDS Model predicts that a *Tyrannosaurus* of leg length 3.1 m and mass 8,000 kg, moving at 5 m s^-1^, would have a duty factor of 0.54 (i.e., it is not airborne), a stance duration of 0.43 s and a stride length of 4.08 m. The vertical component of the GRF would have a gently double-peaked force-time profile, with a peak vertical force of about 117 kN (~1.5 body weights) experienced at 31% of the stance. The whole-body COM movements would have been largely bouncing-like, with percent congruity approximately 90%.

The question may be raised as to how viable it is to extrapolate from the bird species studied here to extinct, non-avian theropods. Modern birds have a distinctly different musculoskeletal anatomy from that of many extinct, non-avian theropods, in terms of limb segment proportions [92,112,113], inferred relative size and positioning of muscular groups[114–116] and inferred limb orientations and COM position [1,20,34,117,118]. Additionally, the absolute range of body masses encompassed by modern birds is small compared to that encompassed by extinct, non-avian theropods. Hence, whilst it may be reasonable to extrapolate to a 200 kg moa, is it reasonable to extrapolate to an eight tonne tyrannosaur?

Whilst there are many important anatomical differences between birds and extinct, non-avian theropods, there are also many important similarities, due to homology. More fundamentally, in the context of terrestrial locomotion birds and non-avian theropods are (or were) obligatory striding, parasagittal bipeds, and so share the same underlying biomechanical requirements [21,46]. No further evidence for this is needed beyond humans. Humans and birds differ considerably in anatomy and posture, and differ in many kinematic and kinetic parameters, including how these parameters change with increasing speed. However, at slow speeds of locomotion, they both employ high duty factors with long stance durations and short stride lengths [21]; the anteroposterior component of the GRF comprises a negative-positive couplet of equal and opposite impulses and the vertical component has two distinct peaks; and the centre of mass moves in such a way that KE and PE oscillate largely out of phase [46]. At fast speeds of locomotion, humans and birds both employ low duty factors with short stance durations and long stride lengths [21]; the anteroposterior component of the GRF comprises a negative-positive couplet of equal and opposite impulses and the vertical component has only a single peak; and the centre of mass moves in such a way that KE and PE oscillate largely in phase [46]. Thus, despite many anatomical and postural differences, humans and birds exhibit gross biomechanical similarity, because they are both obligatory striding, parasagittal bipeds. One further line of evidence supporting the use of birds in making inferences about non-avian theropod locomotion is given by the results of Bishop et al. [43]. The findings of that study suggest that most extinct, non-avian theropods may have had a continuous locomotor repertoire, much like birds.

Anatomical differences aside, the concern about size differences is a legitimate one, and it is difficult to ascertain *a priori* the extent to which extrapolations may be made within an acceptable margin of error. Only through biomechanical modelling, using other data and constraints as inputs, can the reasonableness of the BIRDS Model’s predictions at large body size be identified, and the model subsequently refined (see also below). It is also worth bearing in mind that *in relative terms*, the range of body masses encompassed by the bird species studied is greater than that between extant birds and extinct non-avian theropods: going from a 45 g painted quail to an 80 kg ostrich is a 1,780-fold increase in mass, whereas going from an 80 kg ostrich to an eight tonne *Tyrannosauru*s is only a 100-fold increase in mass. That is, a large part of the relative increase in body mass across the range of theropods is actually covered in the present study.

### GRF asymmetry and the effect of COM location

**Fig 16:**
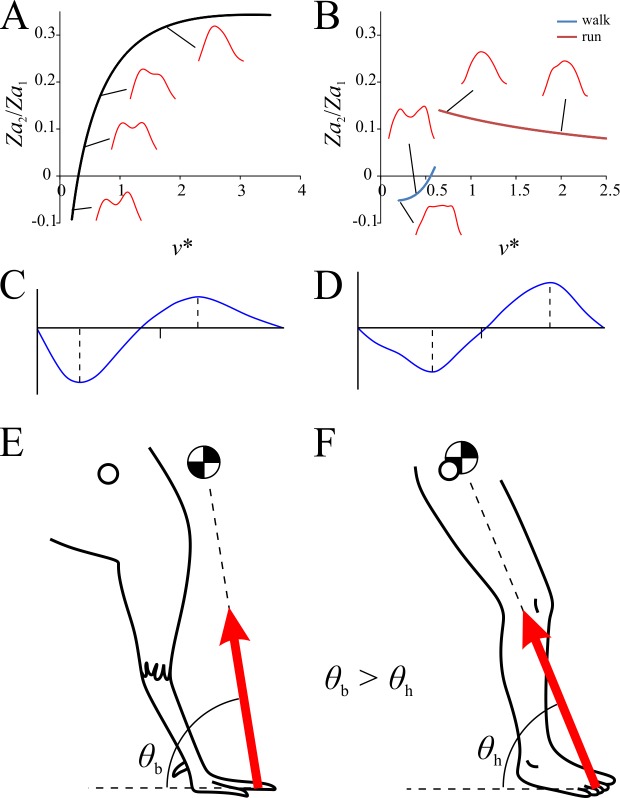
Asymmetry in the force-time profiles of the different components of the GRF. (A, B) Differences in *F*_*z*_ force-time profile asymmetry between birds (A) and humans (B), as quantified by the ratio of Fourier coefficients *Za*_2_/*Za*_1_. (C, D) The force-time profile of *F*_*x*_ exhibits different gross shapes in birds (C) and humans (D). In birds, *t*(*F*_*x*_ = 0), *t*(*F*_*x*,peak_^+^) and *t*(*F*_*x*,peak_^–^) all occur earlier in the stance compared to humans. (E, F) The differences in asymmetry of the *F*_*x*_ force-time profile are probably due to differences in the location of the COM (black and white disk) relative to the hips (hollow circle). As the GRF vector tracks the COM, at *temporally equivalent* points in the stance the GRF vector will be more anteriorly inclined in birds (E) than humans (F). Note that the asymmetry results for a portion of the bird data investigated in this study have previously been reported [64]. However, these results were presented in a preliminary fashion, in raw format, not as the derived predictive relationships presented here.

A further notable difference between birds and humans observed in this study is the nature of asymmetry in the GRF force-time profiles (Fig 7). Except at very slow speeds, the force-time profile of *F*_*z*_ in birds exhibits ‘early-skew’, with the profile tending to be positively skewed along the time axis, and the instance of *F*_*z*,peak_ usually occurring well before mid-stance. There were comparatively few bird trials in which the instance of *F*_*z*,peak_ occurred within 0.4–0.6 of *t*_stance_ (20.5% of trials), across the entire range of speeds. The force-time profile of *F*_*z*_ in humans was far more symmetrical, with the instance of *F*_*z*,peak_ occurring closer to mid-stance; in running, 87.4% of trials had *F*_*z*,peak_ occurring within 0.4–0.6 of *t*_stance_. Asymmetry in the *F*_*z*_ force-time profile may be assessed by calculating the ratio of two Fourier coefficients, *Za*_1_ and *Za*_2_ [64,72,73]. *Za*_1_, of frequency 1 Hz, is the primary determinant of profile magnitude, whereas *Za*_2_, of frequency 2 Hz, is the primary determinant of asymmetry, since it adds to one end of the profile and subtracts away from the other end. The ratio *Za*_2_/*Za*_1_ is therefore a magnitude-normalized measure of profile asymmetry (Fig 16A and 16B; [72]). Negative values of this ratio cause the *F*_*z*_ force-time profile to be late-skewed (when the second peak is larger than the first), as is observed at low speeds; positive values cause the profile to be early-skewed. The degree of asymmetry in the *F*_*z*_ force-time profile (equal to the absolute value of *Za*_2_/*Za*_1_) was larger in birds than in humans at all speeds except in the small range of 0.25 ≤ v* ≤ 0.4; above v* = 1 it was considerably larger in birds.

Differences also existed between birds and humans in the asymmetry of the *F*_x_ force-time profile. As described above, the *F*_*x*_ profile in birds was markedly asymmetric such that *t*(*F*_*x*_ = 0) was almost always less than 0.5, and often significantly so. In contrast, the *F*_*x*_ profile was less asymmetric in humans, with *t*(*F*_*x*_ = 0) almost always greater than 0.5, but only by a small amount (Fig 16C and 16D). Additionally, the instances of *F*_*x*,peak_^+^ and negative *F*_*x*,peak_^−^tended to occur earlier in the stance in birds compared to humans (Figs 4A–4D, 16C and 16D).

The above differences in force-time profile asymmetry between birds and humans possibly reflect differences in the location of the COM relative to the hips. In all bird species studied here, and indeed the majority of extant birds, the COM is considerably anterior to the hip, whereas in humans it is much closer to the hip. This difference was recently proposed by Andrada et al. [119] to be one possible factor responsible for the distinct early-skew observed in the vertical component of the GRF, and has been supported by the experimental findings of Clemente et al. [64]. A theoretical model developed by Andrada and coworkers [119], when inputted with realistic anatomical and gait data, including COM location, can simulate early-skewed *F*_*z*_ force-time profiles reasonably well; however, their model did not predict the late-skewed *F*_*z*_ profiles that were observed at very slow speeds in the present study. Although COM location may influence vertical force-time profile asymmetry, other factors may also contribute, such as intrinsic limb dampening [61] or hip torque-driven limb propulsion [119]. The relative importance of each factor, and whether this changes with body size or speed, requires future study.

The difference in the *F*_*x*_ force-time profile asymmetry between birds and humans may also be potentially reconciled with COM location. In the absence of any moments applied at the feet and about the mediolateral or anteroposterior axes, Newton’s second and third laws dictate that the GRF vector must track the instantaneous location of the whole-body COM throughout the stance phase. Thus, at a given point in the stance, the GRF vector will be more anteriorly oriented in a biped with a more anteriorly located COM, compared to a biped with a more posteriorly located COM (Fig 16E and 16F). As such, the instance of *F*_*x*_ = 0 will occur earlier in the stance phase for the biped with the anteriorly located COM. In other words, in a biped with a more anteriorly located COM, the COM will pass over the centre of pressure of the foot earlier on in the stance phase, such that the GRF vector will be vertical in the sagittal plane earlier on.

### The effect of body mass

The observations reported here, in concert with those reported previously [21,24,36,37], demonstrate that body mass potentially modulates the relationship between at least three important kinematic parameters and speed:

1. Larger birds tend to use lower duty factors at a given relative speed, and in turn, they tend to transition to an aerial run (*β* decreases below 0.5) at lower relative speeds.
2. Larger birds tend to have shorter stance durations at a given relative speed (Fig 2C).
3. Larger birds tend to use lower relative stride lengths at a given relative speed, and in turn, they tend to transition to an aerial run at lower relative stride lengths (Fig 2E).

It is possible that it is not body mass *per se* that modulates these features, but rather it is the degree of crouch, which co-varies with body mass (Fig 1E). Specifically, smaller birds have a more crouched posture, such that the functional length of their leg is proportionally greater than their hip height, allowing them to maintain ground contact with the feet for longer durations (which increases stability), allowing them to make longer relative strides, and allowing them to transition to an aerial run at faster relative speeds [21,120]. This realization has two important implications for how the fossil trackways of theropods, and possibly all bipedal dinosaurs, are interpreted.

Firstly, size-related differences in postural crouch have not been taken into consideration in previous attempts at estimating trackmaker hip height from footprint length [80]. Current methods may hence overestimate hip height in small species, or underestimate hip height in large species, or both; a revision in light of the new empirical data presented here is therefore warranted. Secondly, size-related differences in kinematics means that one simply cannot measure stride length, estimate relative stride length and then immediately identify what ‘gait’ the animal was using [80] (see also [1]), unless the relative stride length was very low (slow ‘walking’, where vaulting-like COM mechanics dominate) or very high (fast ‘aerial running’, where bouncing-like COM mechanics dominate). Moreover, given that birds, and possibly also non-avian theropods, have a continuous locomotor repertoire [43], this further complicates the matter, because ‘walking’ and ‘running’ are not easily distinguished at intermediate speeds.

### Implications for biomechanical modelling

A key aim of this study was to produce predictive equations that may be used to facilitate improved attempts at biomechanical modelling of extinct theropod locomotion. In addition to achieving this aim, two findings of this study have important implications for future biomechanical modelling work.

Of all the variables found to vary significantly with increasing speed in birds, not one concerned the mediolateral component of the GRF, *F*_*y*_. This is despite the fact that in many birds step width (the mediolateral or transverse displacement between successive footfalls) decreases with increasing speed [43]. Consequently, *F*_*y*_ could not be predicted for birds, and thus prediction for extinct theropods cannot be made at the current time. This is not overly problematic, however, since *F*_*y*_ was only found to be a small component of the overall GRF vector (see above), and moreover would be expected to contribute minimally to mechanical energy fluctuations of the whole-body COM. The lack of predictability in *F*_*y*_ possibly reflects the fact that the main movements for a parasagittal biped (such as a typical ground-dwelling bird) are in the sagittal plane, and by extension so too are the forces; it may also reflect to some degree of noise in the experimental data, but how much remains to be determined. It is posited here that in straight-line locomotion, mediolateral forces are probably only (or at least predominantly) exerted for stabilization purposes. That is, they reflect small-scale, step-to-step adjustments made by the bird in order to maintain dynamic stability. Therefore, rather than being an input to a biomechanical model of theropod locomotion, *F*_*y*_ may be viewed as a constraint: simply apply whatever *F*_*y*_ is necessary at each instant in time to maintain dynamic stability.

There is a theoretical precedent to expect that COM location may exert an influence on the nature of the GRF, in particular, the degree of asymmetry in the force-time profiles of *F*_*x*_ and *F*_*z*_. This has important implications for how the BIRDS Model developed here is applied to extinct theropods. The model would need no modification if it were applied to extinct birds, providing that it could be shown that their COM was similarly positioned as in modern birds. However, most (if not all) non-avian theropods are inferred to have had a COM located markedly more posteriorly than in modern birds, closer to the hips [34,82–85]. This would likely limit the application of the model in its current form. Further experimental investigation is required to understand exactly how COM position may influence the GRF. One avenue is comparative studies across related species that employ different postures (e.g., mallard versus Indian runner ducks; [121]), or within species that display anatomical differences due to sex or breeding status (e.g., peafowl; [122]). Experimental studies that artificially manipulate COM location (e.g., [31]) may also offer some improved insight on this matter. Alternatively, humans are also amenable to manipulative studies, and could be subject to experiments where the COM location is altered in a controlled manner (cf. [123–125]). If a systematic relationship was found between force-time profile asymmetry and COM location, then an adjustment could be made to the model to facilitate its application to extinct non-avian theropods, once their COM location was estimated.

## Conclusion

Through the comparative analysis across speed and body size, the results of this study have reiterated the fact that ground-dwelling birds use a highly continuous terrestrial locomotor repertoire. Unlike humans and many other terrestrial animals that have been investigated to date, birds exhibit a continuous change with respect to speed in terms of kinematics. This continuous pattern has now also been demonstrated to occur for ground reaction forces and COM energy fluctuations.

The comparative analysis undertaken here has also facilitated the development of a set of predictive equations that relate several important kinematic and kinetic (GRF) variables to speed in avian terrestrial locomotion, which may be extrapolated to extinct avian and non-avian theropods.

Previously, it had not been possible to empirically predict even basic kinematic or kinetic aspects of locomotion in extinct theropods, yet with the BIRDS Model that comprises these predictive equations, such capabilities are now available, albeit with caveats. The BIRDS Model that comprises these predictive equations has considerable explanatory power, and moreover may be extrapolated to extinct theropods as well. As the location of the COM relative to the hips probably exerts an important influence on the nature of the GRF, this is not the final iteration of the model. Nevertheless, it is an important advancement in facilitating a better understanding of how extinct theropods may have moved around.

The results of this study have also provided further quantitative evidence for the importance of body size on theropod locomotor biomechanics. In particular, body size exerts an influence on the degree of postural crouch, as well as the relationship between speed and kinematic parameters. Thus, at equivalent relative speeds, a small theropod will be moving in a decidedly different fashion to a large theropod. This in turn raises concerns about the accuracy with which palaeontologists can reliably interpret fossil theropod footprints, in terms of inferring the posture and ‘gait’ of the trackmaker.

## Supporting information

S1 Appendix Detailed description of methods and BIRDS model validation. (DOC)

S1 Code MATLAB script that runs the BIRDS model calculations, given user-defined inputs. (TXT)

S1 Spreadsheet Excel spreadsheet listing results from kinematic and kinetic analysis of bird and human data. (XLSX)
