# Supplementary material for: The influence of speed and size on avian terrestrial locomotor biomechanics: Predicting locomotion in extinct theropod dinosaurs
Source: PLoS One. 2018 Feb 21;13(2):e0192172. doi: 10.1371/journal.pone.0192172 (PMC5821450; doi:10.1371/journal.pone.0192172)
Supplement: S1 Appendix — (DOC) [file pone.0192172.s001.doc]

Supporting Information (S1 Appendix) for

**The Influence of Speed and Size on Avian Terrestrial Locomotor Biomechanics: Predicting Locomotion in Extinct Theropod Dinosaurs.**

P.J. Bishop^*^, D.F. Graham, L.P. Lamas, J.R. Hutchinson, J. Rubenson, J.A. Hancock, R.S. Wilson, S.A. Hocknull, R.S. Barrett, D.G. Lloyd and C.J. Clemente.

*Corresponding author: peter.bishop@qm.qld.gov.au

Contents:

1. Detailed methods
2. BIRDS Model validation results
3. References
4. Figures
5. Tables

**Detailed methods**

The outline of the methodology given in the main text of the paper is expanded upon here.

***1. Bird data***

1.1 Data collection

In the present study, a small indoor racetrack was used for studying the three quail species, and a larger outdoor one was used for the remaining species . The small racetrack (Fig S1A) consisted of a 3 × 0.4 m elevated wooden racetrack, with dark ‘hiding places’ at either end. The racetrack was walled with plywood except part of its lateral side, which was replaced with either clear acrylic (for lateral-anterior camera positions) or fine wire mesh (for anterolateral-posterolateral camera positions). The floor of the racetrack was covered with fine grit sandpaper to reduce slippage. A custom-built forceplate was mounted in the middle of the racetrack, covered with fine grit sandpaper and flush with the surrounding racetrack surface. The forceplate comprised a 50 × 50 × 10 mm or 100 × 100 × 10 mm plastic top plate secured to a six degree-of-freedom force-torque sensor (ATI nano17; ATI Industrial Automation, Apex, North Carolina, United States of America).

The large racetrack (Fig S1B) consisted of an 11 × 1 m strip of open ground, which was walled on one side by a large shed, and on the other sides by opaque garden plastic. In the middle part of the lateral side of the racetrack, where the birds were filmed, the opaque plastic was replaced by fine wire mesh. Each end of the racetrack had ‘hiding boxes’ and shade. The top of the racetrack was covered with bird netting, to prevent escape of the birds. One of two larger custom-built forceplates was also mounted in the middle of the racetrack, covered with coarse grit sandpaper and flush with the surrounding racetrack surface. The first one, used for the majority of data collection, has been described previously ; its top plate measured 280 × 280 × 10 mm. The second forceplate, used only in the collection of a portion of the guineafowl data, comprised a six degree-of-freedom force-torque sensor (ATI gamma; ATI Industrial Automation) with a 140 × 140 × 10 mm plastic top plate. The immediate surroundings of both forceplates and their associated electronics were covered by a plywood board and carpet during use; the remainder of the racetrack floor was covered by dirt and gravel.

For all forceplates, the *x*-axis was parallel to the long-axis of the racetrack (i.e., the general direction of travel), the *y*-axis was transverse to this and the *z*-axis was vertical. The natural unloaded frequencies of each forceplate used were greater than 100 Hz in all three directions, far exceeding the maximum stride frequencies observed (< 7 Hz).

Birds were filmed at a 1280 × 1024 pixel resolution, and at a framerate of usually 250 frames second^-1^. A very small fraction (~6%) of trials were inadvertently filmed at 50–150 frames second^-1^ due to equipment error, but since these trials all pertained to larger bird species which moved at lower stride frequencies, this was of no concern. In the large racetrack setup, the cameras were oriented anterolaterally and posterolaterally relative to the direction of travel, and their orientations relative to one another were separated by at least 60°. A similar set of camera orientations was used for filming the painted quail in the small racetrack set up, although here the camera separations more closely approached 90°. For the Japanese and bobwhite quail, the cameras were oriented anteriorly and laterally to the direction of travel, with the anterior camera placed at one end of the racetrack. The forceplates were operated through an analogue-digital converter (NI USB-6343, National Instruments, Austin, Texas, United States of America), which sampled data at 10 kHz using a custom LabVIEW 2012 script (National Instruments). Forceplates were re-zeroed frequently throughout each day of data collection, and the large forceplate was also recalibrated throughout when necessary. Cameras were synchronized with each other and the forceplates using a manual trigger pulse.

For both racetracks, 3-D wooden frames with markers of known coordinates were used to establish a calibration volume for each day’s trials, using an 11-coefficient direct linear transform . The calibration volume for the small racetrack measured approximately 80 × 30 × 20 cm; the calibration volume for the large racetrack measured approximately 140 × 100 × 60 cm. The coordinate system of the calibration volume was parallel to the coordinate system of the forceplates.

1.2 Kinematic data processing

Only trials in which the bird was moving in a relatively straight line, and in which only a single foot (and the entire foot) was placed on the forceplate, were kept for subsequent data processing and analysis. Trials in which the bird displayed obviously abnormal behaviour (e.g., hopping, skipping, alternating pace lengths or sudden lurching to one side) were excluded from analysis. For each trial recorded in the current study, the back and toe markers were digitized and their 3-D coordinates calculated using DLTdv5 , a digitizing program written for MATLAB (version 8.0, MathWorks, Natick, Massachusetts, United States of America). The toe markers were digitized at both the instance of foot touchdown and when the feet were firmly planted on the ground and fully stationary. For the nine species of bird run in the present study, the instance of touchdown or liftoff of a given foot was taken to be the first video frame in which the end of digit III touched the ground, or the last frame that the end of digit III touched the ground, respectively. This is appropriate, for the end of digit III (claw or base of toe) was typically the first part of the foot to make contact with the ground, and typically was the last part to leave the ground. Furthermore, by processing the video and GRF data vis-à-vis, it was observed that touchdown and liftoff as determined from the 250 Hz videos always coincided (visually) very closely to the beginning and end of the vertical force component of the 10,000 Hz GRF measured by the forceplates. The stance duration, *t*_stance_, was subsequently calculated as the time difference between foot touchdown and liftoff.

In all species’ trials, except those of the tinamous, the direction of heading of the bird as it crossed the forceplate was determined by fitting a least-squares straight line to the (*x*,*y*) coordinates of the trajectory of the back markers. As the tinamous were only filmed in lateral view, they were assumed to be moving parallel to the forceplate . The Cartesian gradient of this line was then used to calculate the angle of the bird’s heading with respect to the *x*-axis of the forceplate (global) coordinate system. If the available trajectory of the back markers was limited in a given trial (for instance, due to inability to completely digitize the trajectory of back markers), then the direction of heading was determined by fitting a least-squares straight line to the (*x*,*y*) coordinates of the digitized coordinates of stationary footfalls. In this case, as many digitized footfalls as possible from the trial were used. At the very minimum, three successive footfalls are necessary: the preceding footfall, the footfall contacting the forceplate and the following footfall. The mean forward speed in the direction of heading, *v*, was also determined for each trial (except for the tinamous, which had previously been measured: ), preferentially from the back marker trajectory if available, but otherwise from the timing and spatial position of foot touchdowns. Comparison of the 588 trials in which both back markers and footfalls were able to be used to calculate heading and speed indicated that the two methods yielded highly comparable values (Fig S2). A major axis linear fit of the speed estimates (with the fitted line forced through the origin) has a slope of 1.024, with an *r*^2^ = 0.981, calculated in PAST 3.09 . These results support the use of the footfalls as an alternative to back marker trajectories when necessary.

Duty factor, *β*, was determined for each trial from the instances of foot touchdown and liftoff. Duty factor is defined as the proportion of the stride for which a given foot (here, the foot contacting the forceplate) is on the ground, that is, the stance duration divided by stride duration. The stride duration may be defined either using the same foot (i.e., that contacting the forceplate), or the other (contralateral) foot, and hence duty factor may be determined in one of three ways (Fig S3A):

$\beta=\frac{LO_{N}-TD_{N}}{TD_{N+2}-TD_{N}}$, (S1)

$\beta=\frac{LO_{N}-TD_{N}}{TD_{N}-TD_{_{N-2}}}$, (S2)

$\beta=\frac{LO_{N}-TD_{N}}{TD_{N+1}-TD_{N-1}}$, (S3)

where *TD_n_* and *LO_n_* signify touchdown and liftoff of footfall *n*, respectively, and {*N*–2, *N*–1, *N*, *N*+1, *N*+2} denote the second preceding footfall (same foot as that which touches the forceplate), preceding footfall (contralateral foot), footfall touching the forceplate, following footfall (contralateral foot) and second following footfall (same foot as that which touches the forceplate). If the bird is moving at a constant speed with exactly equal phase relationship of the feet, then all three methods of calculation will yield the same result. This is not always the case, and so equation S3 was used preferentially here wherever possible, as it acts to ‘average out’ unevenness in step-to-step phase (as well as speed), by including both a footfall before and after that which contacts the plate. Whenever the situation did not permit equation S3 to be used, then either equation S1 or S2 was used instead. In reality, the results produced by all three methods always eventuated to be very similar for a given trial, as there was typically only a small difference in phase between successive footfalls. If there were only two footfalls recorded in the field of view of the cameras, the duty factor was determined assuming that both feet have equal duty factors and phase relationships. This requires the calculation of a ‘virtual third touchdown’, that is, when the third foot would have touched down if it had indeed been recorded (Fig S3B):

$\beta=\frac{LO_{1}-TD_{1}}{TD_{3}-TD_{1}}=\frac{LO_{1}-TD_{1}}{LO_{2}-LO_{1}+TD_{2}-TD_{1}}$. (S4)

An analogous calculation was used if it was footfall 2 that contacted the plate (cf. Fig S3B; the denominator remains unchanged, however).

For the birds run in the current study, duty factor was determined from the instances of touchdown and liftoff as identified in the videos. For the ostriches, touchdown and liftoff was identified from visual inspection of the temporal variation of the vertical (*z*) coordinate of the toe marker. As with the birds run in the present study, there was close visual agreement between this kinematic and recorded force data in terms of the instances of foot touchdown and liftoff. For the emu and tinamou data, touchdown and liftoff were identified from the vertical component of the raw GRF data only. The time of touchdown was taken to be when the vertical force exceeded the background ‘noise’ of the unloaded forceplate; the time of liftoff was taken to be when the vertical force no longer exceeded the background 'noise' of the unloaded forceplate. Owing to the lower sampling frequencies used for tinamous and emus , these points could be easily identified by visual inspection with negligible error. Whilst the precise method used for calculating duty factor differed between species, depending on the availability of kinematic and GRF data for footfalls preceding and following that which contacted the forceplate, potential error due to differences in methods are considered to be minimal. This is because of the high frequency of data recording compared to the birds’ stride frequencies; errors on the order of even 5 frames at a 250 frames second^-1^ recording frequency amount to only 2%.

The stride length *S* in each trial was determined as the length of the stride opposite the foot contacting the plate (i.e., measured from footfall *N*–1 to footfall *N* +1). Two exceptions to this were the data for tinamous (in which stride length had been previously measured: ), and emus, in which only the right digit III had been marked and kinematically tracked . As such, the stride length could not be measured directly; instead, it was estimated using the method described by Bishop et al. .

1.3 GRF data processing

For the large forceplate described previously , raw voltage recordings were processed in a custom MATLAB script. Briefly, the recorded voltages were synchronized to the videos. Once trimmed to foot touchdown and liftoff, the voltage recording for each of the six channels were baselined and corrected for any plasticity effects that occurred during the stance, assuming a linear response. Each channel was then filtered with a fourth-order, zero-lag Butterworth low-pass filter (a buffer zone of data before touchdown and liftoff was retained for this step). The cutoff frequencies used for each channel removed noise without attenuating signal peaks, and were chosen based on visual comparison of the filtered *versus* unfiltered data; they varied largely between 20–35 Hz. The filtered voltages were then converted to forces . These forces were corrected for the direction of heading of the bird, so as to have them expressed in the bird’s anatomical coordinate system. Lastly, the sign of the *x* and *y* components of the forces were adjusted as necessary, in order to maintain a consistent anatomical convention: +*x* is forward, + *y* is medial and +*z* is upwards.

A similar process was employed in the processing of the force data recorded by the other forceplates for the quail and guineafowl, as well as for the previously collected tinamou, emu and ostrich data. The processing of data collected with the small racetrack forceplate included a custom calibration routine, as one of the 6 factory channels in the ATI nano17 force transducer was found to be unreliable. The cutoff frequencies used for the quail varied largely between 50–80 Hz (Japanese and bobwhite quail) or between 60–100 Hz (painted quail); for the guineafowl with the ATI gamma force transducer largely between 30–50 Hz; for the tinamous between 70–90 Hz; and for the emus between 15–20 Hz. The ostrich force data had previously already been Butterworth filtered by at 15 Hz .

***2. Human data***

Prior to data collection, each subject’s natural walk-run transition speed was ascertained by starting them on the treadmill at a slow walking speed and gradually increasing the tread speed until they spontaneously switched to a run. This transition speed was validated by increasing the tread speed further, and then gradually decreasing the speed until the subject spontaneously switched back to a walk. For each subject, their walk-run and run-walk transition speeds were approximately the same.

For each walking and running trial, the subject initially stood still on the stationary tread, whence the tread speed was gradually increased to the desired trial speed over a period of 5–10 s. After the subject had reached a steady manner of locomotion, data recording commenced; a minimum of four strides were recorded at each trial speed. Subsequently, the tread speed was slowly reduced back to zero. The order of trial speeds was randomized, and the subjects did not know what the trial speed was going to be. For each trial the subjects were asked to use their preferred manner of locomotion (walking or running). One exception to this was the subject-specific walk-run transition speed, which was tested twice. In the first trial, the subject used their naturally preferred gait; in the second trial, they were instructed to use the other gait. That is, if the first trial was a walk, then in the second trial they were instructed to run, and vice versa. After each trial, the force sensors in the treadmill were re-zeroed, and the subject was allowed to rest between trials as required.

A potential caveat of the current study is the comparison of data from overground locomotion in birds to that collected from treadmill locomotion in humans. The use of the treadmill was chosen so as to facilitate the acquisition of a large amount of data in a short time span, and also because it was already set up and calibrated with the motion capture system; an overground racetrack of suitable length required for this study, with calibrated cameras, was not available during the period of data collection. Several previous studies have demonstrated that the GRFs recorded for human locomotion on a treadmill do not substantially differ, qualitatively or quantitatively, from that recorded for overground locomotion . Indeed, the data collected in this study are quite comparable to that reported previously for overground walking and running in humans . As the human data were collected solely for gross comparative purposes birds, the difference in data collection methods was therefore deemed inconsequential for the present study.

***3. GRF analysis***

3.1 Mechanical energy fluctuations

Usually, the GRFs from an integral number of strides are required to examine patterns of mechanical energy fluctuation . However, if *β* is known, then the total GRF profile experienced throughout the course of a whole stride can be simulated, by superimposing the recorded single-footfall GRF profile on itself and incorporating a ‘phase shift’ for the temporal offset of right and left footfalls (Fig S4). This procedure assumes that the subject is moving at a steady speed with left-right symmetry in limb use (e.g., equal duty factors and footfall phase relationships), which is a good first approximation but may not always be exactly true . The start of the stride is designated to be the touchdown of the foot that contacts the forceplate.

As *β* and *t*_stance_ were already known, the stride duration was calculated as

$t_{\text{stride}}=\frac{t_{\text{stance}}}{\beta}$. (S5)

In a symmetrical gait, as is assumed here, the phase shift of the GRF profile from the contralateral foot is half of the stride duration, regardless of duty factor:

$P=\frac{t_{\text{stride}}}{2}$, or (S6)

$P=\frac{t_{\text{stance}}}{2\beta}$. (S7)

Thus, the original GRF profile is superimposed upon itself, being separated by a phase shift of *t*_stance_/2*β*, although the profile of the mediolateral component is reversed about the abscissa (i.e., multiplied by -1), since the opposite leg will push with the mediolaterally opposite pattern. The two GRF profiles are then summed to produce the total GRF profile across the whole stride.

Fluctuations of KE and PE were then calculated via single and double integration of the total force profile, as follows. Instantaneous accelerations were determined thus:

$a_{x}=\frac{F_{x}}{m}$, (S8)

$a_{y}=\frac{F_{y}}{m}$, (S9)

$a_{z}=\frac{(F_{z}-mg)}{m}$, (S10)

where *m* is body mass and *g* is the acceleration due to gravity, 9.81 m s^-2^. Instantaneous velocities were determined thus:

$v_{x}=\int_{0}^{t} a_{x}dt+u_{x}$, (S11)

$v_{y}=\int_{0}^{t} a_{y}dt$, (S12)

$v_{z}=\int_{0}^{t} a_{z}dt+u_{z}$. (S13)

Instantaneous vertical displacement was determined thus:

$s_{z}=\int_{0}^{t} v_{z}dt$. (S14)

Instantaneous kinetic energy was determined thus:

. (S15)

Instantaneous gravitational potential energy was determined thus:

$\text{PE}=mgs_{z}$. (S16)

In the calculations of instantaneous velocities, *u_x_* is the mean forward velocity of the bird, as measured from kinematics. The pronumeral *u_z_* is the vertical velocity of the centre of mass at the beginning of the stride . This is non-zero because in steady-state locomotion, the overall vertical displacement of the centre of mass over a whole stride is zero:

$\int_{0}^{t_{\text{stride}}} v_{z}dt=0$. (S17)

Given equation S13, this implies that

$\int\int_{0}^{t_{\text{stride}}} a_{z}dt+\int_{0}^{t_{\text{strid}e}} u_{z}dt=0$, (S18)

$\int\int_{0}^{t_{\text{stride}}} a_{z}dt+ u_{z}t_{\text{stride}}=0$, and therefore (S19)

$u_{z}=-\int\int_{0}^{t_{\text{stride}}} a_{z}dt\div t_{\text{stride}}$. (S20)

As $\int\int_{0}^{t_{\text{stride}}} a_{Z}dt$ is non-zero, so too will be the value for *u_z_*. Thus, the value of *u_z_* was determined by requiring the overall vertical displacement of the COM over a whole stride is zero, or equivalently, that the average vertical velocity of the COM over a whole stride is zero . The calculation of instantaneous mediolateral velocity (equation S12) did not require an integration constant, since the subject moved in a straight line and thus there was no net mediolateral displacement. The calculation of instantaneous vertical displacement (equation S14), in not having an integration constant, ignored the PE already present by virtue of the height of the COM above the ground; rather, it assessed how PE changed relative to a datum, in this case, the PE of the COM at the beginning of the stride.

3.2 Fourier analysis

The process of Fourier analysis of the force-time profile of each of the three components of the GRF was run in a custom MATLAB script, as follows:

1. The force-time profiles of each component were normalized to body weight and stance duration, that is,

$F*=\frac{F}{mg}$, (S21)

$t*=\frac{t}{t_{\text{stance}}}$. (S22)

1. A full-wave signal was created by duplicating the force-time profile, reversing it with respect to both force and time and appending it to the end of the original force-time profile (Fig S5). Such a transformation ensures that the transition from the original force-time trace to the transformed trace is smooth . This also minimizes ‘spectral leakage’, where higher frequencies are recovered with artificially inflated values of their respective coefficients *a_n_* or *b_n_*.
2. A fast Fourier transform was applied, and coefficients *a_n_* and *b_n_* were calculated.
3. Additionally, a power spectrum of the coefficients was calculated for each component of the GRF, beginning at frequency 1Hz and progressing towards higher frequencies, to determine the relative contribution of each sine and cosine term towards explaining the shape of the force-time profile.

As the fast Fourier transform was applied to the full-wave signal, the vertical offset of the signal is zero, that is, *a*_0_ = 0. Hence, the recovered values for coefficients *a_n_* and *b_n_* reflect the shape of the curve only, and conversely, the entire shape of the curve is described by those coefficients.

***4. Statistical analyses***

Before the results were subject to statistical analysis, trials were discriminated according to three criteria, to identify ‘good’ steady-state trials. These criteria were:

1. Excess imbalance in forwards *versus* backward impulses of *F_x_* was ≤ 150%.
2. Relative speed change across the duration of stance (as calculated from the net impulse in *F_x_*) was ≤ 10%.
3. The dynamic adjustment factor applied (*α*) was between 0.8–1.2.

After applying these three criteria, a total of 701 bird trials and 243 human trials were found to be satisfactory for statistical analysis (S1 spreadsheet).

As the primary objective of this study is prediction, it was imperative to maximize the generality of the predictive equations derived, even if this entailed sacrificing some fine-scale accuracy concerning the effects of body mass or phylogeny. To initially identify which variables varied significantly with relative speed in birds, an ordinary least squares linear regression (*y* = *Ax* + *B*) was conducted for each variable for each species, and significance levels calculated. A majority-rules consensus over the 12 species determined whether a given variable was to be included or not in further analysis. Of those variables identified as significantly varying with relative speed, two further regressions were conducted for each species’ data sets: a simple power fit (*y* = *Ax^B^*, ‘power I’) and a vertically translated power fit (*y* = *Ax^B^* + *C*, ‘power II’). In the determination of the power II fits, the value for *C* was fixed for each species, taken as the value of *C* when a power II fit was applied to the pooled data for all species. By restricting the number of unknown coefficients to two (*A* and *B*), this eliminates the potential for spurious results in nonlinear regression, resulting from multiple local minima in the sum of squared residuals .

Deciding whether a linear, power I or power II fit best explained the data for a given variable primarily used the Akaike Information Criterion (AIC), applying a majority-rules consensus over the 12 species. Thus, a single type of fit was identified to best explain a given variable, and for each species the values of the coefficients *A* and *B* differed.

To assess whether there was a significant influence of body mass on the relationship between a given variable and relative speed, a phylogenetic generalized least squares (PGLS) linear fit was calculated for both *A* and *B* against log_10_*m*, conducted using the ‘caper’ package in R . Body mass for each species was taken as the average of the masses of each individual of that species, weighted by the relative contribution of each individual’s trials to the species’ dataset. To assess the strength of phylogenetic signal on the values of *A* or *B*, the *K* statistic and corresponding *P*-value of was also calculated, using the ‘phytools’ package in R . If it was found that there was no significant influence of mass on the value of a coefficient (*A* or *B*), then the mean value of that coefficient across species was used in the predictive equation. Further, if it was also found that there was no significant influence of phylogeny on the value of *A* and *B* for a given variable, then a single all-species fit was performed to derive the predictive relationship for that variable. This is because without any significant effect of body mass or phylogeny, their data can be pooled together to produce the most generalized predictive equation.

The PGLS fits and calculations of the *K* statistic were based on a consensus phylogeny drawn from the results of recent studies of avian interrelationships (Fig S6, Table S1; ). The branch lengths of the phylogeny used in the statistical analyses are of time since divergence, in millions of years; these were determined as the mean values from each study where such divergence dates were reported (Table S1).

For some variables, it was apparent upon visual inspection that the predictive equation (or equations, if there was an effect of mass on *A* or *B*) yielded by the approach outlined above was unsatisfactory at high or low values of mass or relative speed, with gross over-prediction or under-prediction (often by 100% or more) observed. Furthermore, in the case of some Fourier coefficients, negative values for *F_z_* were predicted towards the end of the stance, which is physically implausible. This error probably stems from at least two factors, the first being the nature of power models. If either the value of *A* or *B* changes from positive to negative (or vice versa), according to mass-dependency identified by PGLS fitting, this will drastically alter the shape of the fitted power curve. The second factor is that whilst the PGLS approach may identify a significant effect of mass on the value of either *A* or *B*, this effect may not apply uniformly across the entire range of body sizes. Consequently, this can lead to erroneous predictions at the extremes of body mass or speed. When such situations were apparent (they were in fact quite obvious), a simpler model was chosen to represent the data (e.g., a linear fit was chosen instead of a power fit), and the coefficients recalculated. Importantly, the AIC values for these simpler models were not greatly different from the AIC values of the ‘best’ model initially identified. In a few cases the use of a simpler model still did not fully remedy the problem of erroneous prediction at the extremes of mass or speed. As such, a single, all-species fit was derived (using the simpler model). Whilst this approach inherently ignores the effects of mass and phylogeny, it was considered appropriate because of the necessity to maximize the generality of the predictive equations derived. Indeed, in those instances where a simpler or more general model was fitted, an (often considerable) increase in *r*^2^ values was almost always observed. Furthermore, the calculated values of the *K* statistic indicated that by and large there was only a small effect of phylogeny on the variables examined.

***5. Model validation***

Theoretically, the BIRDS Model derived in this study can predict a number of important kinematic variables and the nature of the GRF throughout the stance, given just two input variables, body mass and speed. To assess the validity of the BIRDS Model, four tests were conducted:

1. For each of the 701 trials analyzed above, both *m* and *v** were known, and these were used to predict the GRF across the stance. This was then compared to the actual measured GRF force-time profile, and *r*^2^ and root mean squared error (RMSE) values were calculated for each trial.
2. For each of the 701 trials analyzed above, the predicted GRF was also used to calculate those parameters which were found to change significantly with increasing *v**. These calculated parameters were then compared to their measured values for each trial, and *r*^2^ and RMSE values were calculated.
3. To test the internal consistency of the kinematic predictions, stride frequency was calculated in two separate ways, from the predictions of duty factor, stance duration and stride length:

$f_{1}=\frac{1}{t_{\text{stride}}}=\frac{\beta}{t_{\text{stance}}}$, (S23)

$f_{2}=\frac{v}{S}$. (S24)

Ideally, the ratio of *f*_1_/*f*_2_ at all speeds is unity.

1. An important point of consideration in using the BIRDS Model for much larger bipeds (e.g., multi-tonne theropods), is how well it performs in extrapolating beyond the mass range of species studied. Hence, to explore the efficacy of the BIRDS Model in extrapolation, its constituent equations were recalculated excluding the ostrich dataset; then, using the recalculated equations, the GRF was predicted for each ostrich trial and compared to the actual measured GRF profile. A similar approach was also used at the lower end of the mass spectrum, by excluding the data for the painted quail and recalculating the equations, and then comparing the predicted GRF against the actual measured GRF for each trial.

**BIRDS Model validation tests**

The results of the model validation testing are described in detail here.

In comparing the model-predicted *F_x_* and *F_z_* force-time profiles against the measured profiles for each trial, this revealed the BIRDS Model to have relatively high *r*^2^ and relatively low RMSE values. On average across the 701 trials, *r*^2^ = 0.688 and RMSE = 0.084 for *F_x_*, and *r*^2^ = 0.833 and RMSE = 0.214 for *F_z_* (when corrections for dynamic consistency were applied). That is, the model could on average explain five-sixths of the variation in the observed vertical component of the GRF and over two-thirds of the variation in the observed anteroposterior component. A comparison of each individual variable describing the GRF force-time profiles as predicted by the model, against those measured for each trial, indicated that the model performs well overall (Fig S7), predicting the general pattern of change with respect to increasing *v**. Moreover, the model also performed well in comparison to the predictive equations derived individually for each variable, in some cases out-performing them (Table S2).

The ratio of the two different estimates of stride frequency as predicted by the BIRDS Model (*f*_1_/*f*_2_) by and large tended to hover a little above unity (Fig S8), suggesting considerable internal consistency in the kinematic component of the model. The greatest discrepancy between the model’s predictions and the observed data (and the ideal value of *f*_1_/*f*2 = 1) occurred at very low relative speeds for small body masses, and high relative speeds for large body masses. In both cases the ratio of *f*_1_/*f*_2_ exceeded unity considerably, suggesting some combination of overestimation of duty factor, underestimation of stance duration or overestimation of stride length. It should be noted, however, that these regions of discrepancy correspond to the two kinds of data for which fewer trials were collected. For example, data for the large birds (emus and ostriches) does not include any trials with *v** > 1.94 (which corresponds to the point that the 80 kg line leaves the data point cloud in Fig S8). Similarly, data for the three quail species includes very few trials with *v** < 0.35 (which corresponds to the point that 0.05 kg line leave the data point cloud in Fig S8). Consequently, with better sampling of data, the model’s internal consistency might be improved further.

The results of the BIRDS Model extrapolation tests are reported in Table S3. As expected, the recalculated (either ostrich-less or quail-less) model performed worse compared to its all-species form, but this is only slight. The error, relative to the all-species model, is about 3% or less.

**References**

1. Bishop PJ, Clemente CJ, Weems RE, Graham DF, Lamas LP, et al. (2017) Using step width to compare locomotor biomechanics between extinct, non-avian theropod dinosaurs and modern obligate bipeds. Journal of the Royal Society Interface 14: 20170276.

2. Clemente CJ, Bishop PJ, Newman N, Hocknull SA (in press) Steady bipedal locomotion with a forward situated whole-body centre of mass: the potential importance of temporally asymmetric ground reaction forces. Journal of Zoology.

3. Hedrick TL (2008) Software techniques for two- and three-dimensional kinematic measurements of biological and biomimetic systems. Bioinspiration & Biomimetics 3: 034001.

4. Hancock JA, Stevens NJ, Biknevicius AR (2007) Whole-body mechanics and kinematics of terrestrial locomotion in the Elegant-crested Tinamou *Eudromia elegans*. Ibis 149: 605-614.

5. Hammer Ø, Harper DAT, and Ryan PD (2001) PAST: Paleontological Statistics Software Package for Education and Data Analysis. Palaeontologia Electronica 4: 4.

6. Lamas LP (2015) Musculoskeletal biomechanics during growth on emu (*Dromaius*; Aves): An integrative experimental and modelling analysis. [PhD]: Royal Veterinary College, University of London.

7. Rubenson J, Lloyd DG, Heliams DB, Besier TF, Fournier PA (2011) Adaptations for economical bipedal running: the effect of limb structure on three-dimensional joint mechanics. Journal of the Royal Society Interface 8: 740-755.

8. Kram R, Griffin TM, Donelan JM, Chang YH (1998) Force treadmill for measuring vertical and horizontal ground reaction forces. Journal of Applied Physiology 85: 764-769.

9. White SC, Yack HJ, Tucker CA, Lin HY (1998) Comparison of vertical ground reaction forces during overground and treadmill walking. Medicine & Science in Sports & Exercise 30: 1537-1542.

10. Riley PO, Paolini G, Croce UD, Paylo KW, Kerrigan DC (2007) A kinematic and kinetic comparison of overground and treadmill walking in healthy subjects. Gait & Posture 26: 17-24.

11. Riley PO, Dicharry J, Franz J, Croce UD, Wilder RP, et al. (2008) A Kinematics and Kinetic Comparison of Overground and Treadmill Running. Medicine & Science in Sports & Exercise 40: 1093-1100.

12. Kluitenberg B, Bredeweg SW, Zijlstra S, Zijlstra W, Buist I (2012) Comparison of vertical ground reaction forces during overground and treadmill running. A validation study. BMC Musculoskeletal Disorders 13: 235.

13. Willems PA, Gosseye TP (2013) Does and instrumented treadmill correctly measure the ground reaction forces? Biology Open 2: 1421-1424.

14. Alexander RM, Jayes AS (1980) Fourier analysis of forces exerted in walking and running. Journal of Biomechanics 13: 383-390.

15. Cavanagh PR, LaFortune MA (1980) Ground reaction forces in distance running. Journal of Biomechanics 13: 397-406.

16. Hamill J, Bates BT, Knutzen KM, Sawhill JA (1983) Variations in ground reaction force parameters at different running speeds. Human Movement Science 2: 47-56.

17. Munro CF, Miller DI, Fuglevand AJ (1987) Ground reaction forces in running: a reexamination. Journal of Biomechanics 20: 147-155.

18. Nilsson J, Thorstensson A (1989) Ground reaction forces at different speeds of human walking and running. Acta Physiologica Scandinavica 136: 217-227.

19. Keller TS, Weisberger AM, Ray JL, Hasan SS, Shiavi RG, et al. (1996) Relationship between vertical ground reaction force and speed during walking, slow jogging, and running. Clinical Biomechanics 11: 253-259.

20. Giarmatzis G, Jonkers I, Wesseling M, Van Rossom S, Verschueren S (2015) Loading of Hip Measured by Hip Contact Forces at Different Speeds of Walking and Running. Journal of Bone and Mineral Research 30: 1431-1440.

21. Cavagna GA (1975) Force plates as ergometers. Journal of Applied Physiology 39: 174-179.

22. Cavagna GA, Heglund NC, Taylor CR (1977) Mechanical work in terrestrial locomotion: two basic mechanisms for minimizing energy expenditure. American Journal of Physiology 233: R243-R261.

23. Heglund NC, Cavagna GA, Taylor CR (1982) Energetics and mechanics of terrestrial locomotion. III. Energy changes of the centre of mass as a function of speed and body size in birds and mammals. Journal of Experimental Biology 79: 41-56.

24. Blickhan R, Full RJ (1992) Mechanical work in terrestrial locomotion. In: Biewener AA, editor. Biomechanics: Structures and Systems. New York: Oxford University Press. pp. 75–96.

25. Andrada E, Haase D, Sutedja Y, Nyakatura JA, Kilbourne BM, et al. (2015) Mixed gaits in small avian terrestrial locomotion. Scientific Reports 5: 13636.

26. Kambic RE, Roberts TJ, Gatesy SM (2015) Guineafowl with a twist: asymmetric limb control in steady bipedal locomotion. Journal of Experimental Biology 218: 3836-3844.

27. Gard SA, Miff SC, Kuo AD (2004) Comparison of kinematic and kineti methods for computing the vertical motion of the body center of mass during walking. Human Movement Science 22: 597-610.

28. Donelan JM, Shipman DW, Kram R, Kuo AD (2004) Mechanical and metabolic requirements for active lateral stabilization in human walking. Journal of Biomechanics 37: 827-835.

29. Motulsky HJ, Ransnas LA (1987) Fitting curves to data using nonlinear regression: a practical and nonmathematical review. FASEB Journal 1: 365-374.

30. Orme D, Freckleton R, Thomas G, Petzoldt T, Fritz S, et al. (2015) caper: Comparative Analyses of Phylogenetics and Evolution in R.

31. Blomberg SP, Garland T, Jr, Ives AR (2003) Testing for phylogenetic signal in comparative data: behavioural traits are more labile. Evolution 57: 717-745.

32. Revell LJ (2012) phytools: an R package for phylogenetic comparative biology (and other things). Methods in Ecology and Evolution 3: 217-223.

33. Hackett SJ, Kimball RT, Reddy S, Bowie RCK, Braun EL, et al. (2008) A Phylogenomic Study of Birds Reveals Their Evolutionary History. Science 320: 1763-1768.

34. Morgan-Richards M, Trewick SA, Bartosch-Härlid A, Kardailsky O, Phillips MJ, et al. (2008) Bird evolution: testing the Metaves clade with six new mitochondrial genomes. BMC Evolutionary Biology 8: 20.

35. Ksepka DT (2009) Broken gears in the avian molecular clock: new phylogenetic analyses support stem galliform status for *Gallinuloides wyomingensis* and rallid affinities for *Amitabha urbsinterdictensis*. Cladistics 25: 173-197.

36. Phillips MJ, Gibb GC, Crimp EA, Penny D (2010) Tinamous and Moa Flock Together: Mitochondrial Genome Sequence Analysis Reveals Independent Losses of Flight among Ratites. Systematic Biology 59: 90-107.

37. Haddrath O, Baker AJ (2012) Multiple nuclear genes and retroposons support vicariance and dispersal of the palaeognaths, and an Early Cretaceous origin of modern birds. Proceedings of the Royal Society of London, Series B 279: 4617-4625.

38. Jetz W, Thomas GH, Joy JB, Hartmann K, Mooers AO (2012) The global diversity of birds in space and time. Nature 491: 444-448.

39. Smith JV, Braun EL, Kimball RT (2013) Ratite Nonmonophyly: Independent Evidence from 40 Novel Loci. Systematic Biology 62: 35-49.

40. Yuri T, Kimball RT, Harshman J, Bowie RCK, Braun MJ, et al. (2013) Parsimony and Model-Based Analyses of Indels in Avian Nuclear Genes Reveal Congruent and Incongruent Phylogenetic Signals. Biology 2: 419-444.

41. Mitchell KJ, Llamas B, Soubrier J, Rawlence NJ, Worthy TH, et al. (2014) Ancient DNA reveals elephant birds and kiwi are sister taxa and clarifies ratite bird evolution. Science 344: 898-900.

42. Jarvis ED, Mirarab S, Aberer AJ, Li B, Houde P, et al. (2014) Whole-genome analyses resolve early branches in the tree of life of modern birds. Science 346: 1320-1331.

43. Ksepka DT, Phillips MJ (2015) Avian diversification patterns across the K-Pg boundary: influence of calibrations, datasets, and model misspecification. Annals of the Missouri Botanic Garden 100: 300-328.

44. Prum RO, Berv JS, Dornburg A, Field DJ, Townsend JP, et al. (2015) A comprehensive phylogeny of birds (Aves) using targeted next-generation DNA sequencing. Nature 526: 569-573.

**Figures**

**Fig S1. Diagrammatic illustration of the experimental setups used to obtain bird kinematic and force data.** (A) Small racetrack used for the three quail species. (B) Large racetrack used for the remaining species.

**Fig S2. Comparison of the two methods used to measure direction of heading and speed.** (A) Direction of heading; dashed lines are lines of positive and negative parity. (B) Speed in the direction of heading; dashed line is line of parity. Inset in A shows how heading angles were defined with respect to the Cartesian coordinate system of the forceplate. Clearly, both methods produce estimates that are in strong agreement with each other.

**Fig S3. Schematic of the definitions used in calculating duty factor.** (A) The definitions used when three or more footfalls were available. (B) The definitions used when only two footfalls were available; in this instance, a ‘virtual third touchdown’ (in grey) must be inferred in order to calculate duty factor. TD is touchdown, LO is liftoff.

**Fig S4. The simulation of the GRF over a whole stride.** This is achieved by superimposing the GRF measured for one foot onto itself with an appropriate temporal offset. (A) For when duty factor > 0.5. (B) For when duty factor = 0.5. (C) For when duty factor > 0.5. In each case, the temporal offset is equal to *t*_stance_/2*β*. Note that the profile for the total force is slightly offset vertically, for ease of visualization.

**Fig S5. Preprocessing a force-time profile for Fourier analysis.** (A) The original profile has been normalized to body weight and stance duration. (B) A full-wave signal is generated by reversing the profile about both the force and time axes, and appending it to the end of the original profile.

**Fig S6. Phylogenetic relationships of the species investigated in this study.** As far as the species investigated here are concerned, there is very strong agreement in their interrelationships, the only discrepancies being that Jetz et al. recovered ostriches as closer to emus than tinamous, and that Ksepka recovered chickens as closer to *Coturnix* quail than to turkeys. Branch lengths are calibrated to time since divergence; for specific nodes, see Table S1.

**Fig S7. Comparison of predictions of the BIRDS Model against the data for ten GRF variables.** (A) *F_x_*_,peak_^+^. (B) *F_x_*_,peak_^–^. (C) *F_z_*_,peak_. (D) *F_z_*_,mean_. (E) *t*(*F_x_*_,peak_^+^). (F) *t*(*F_x_*_,peak_^–^). (G) *t*(*F_x_*=0). (H) *F_z_*(*F_x_*=0). (I) *F_x_*_,MS_. (J) *F_z_*_,MS_. Qualitatively, the model’s predictions (red line) generally fit the observed data well. Corresponding *r*^2^ and RMSE values for each comparison are reported in Table S2.

**Fig S8. Comparison of predictions of the BIRDS Model against the observed data for the ratio of stride frequency estimates, *f*_1_/*f*_2_.**

**Tables**

**Table S1. Divergence dates of each node in the phylogeny used in the statistical analyses.** See Fig S6 for node locations.

| **Node** | **Date of divergence given by each study,**  **in millions of years ago (mya)** | | | | | | | **Mean dates, mya** |
| --- | --- | --- | --- | --- | --- | --- | --- | --- |
|  | (1) | (2) | (3) | (4) | (5) | (6) | (7) |  |
| 1 (Neornithes) | 97.3 | 127 | 113.2 | 101.6 | 101 | 111.9 | 72.9 | 103.6 |
| 2 (Palaeognathae) | 83.4 | 97 | 85.9 | 83.8 | 72 | 76.8 | 50.7 | 78.5 |
| 3 | 70.6 | 81 | n/a* |  | 65.3 | 66.6 | 39.3 | 64.6 |
| 4 (Neognathae) |  | 106 | 97.6 | 88.6 | 88.9 | 97.8 | 71.9 | 91.8 |
| 5 (Galliformes) |  | 83 | 56.6 |  | 57.5 | 49.4 | 46.2 | 58.5 |
| 6 |  |  | 27.3 |  |  | 29.9 | 28.8 | 28.7 |
| 7 |  |  | 26.4 |  |  |  | 26 | 26.2 |
| 8 (Phasianidae) |  |  | 18.8 |  |  | 18.7 |  | 18.8 |
| 9 |  |  | 18.1 |  |  |  |  | 18.1 |
| 10 (*Coturnix*) |  |  | 0.52 |  |  |  |  | 0.52 |
| 11 |  |  | 71.5 | 66.8 |  | 68.3 | 65.7 | 68.1 |

(1) = ref.

(2) = ref.

(3) = ref.

(4) = ref.

(5) = ref.

(6) = ref.

(7) = ref.

* = date not possible, because in that study a different topology for palaeognaths was recovered.

**Table S2. The predictive performance of the BIRDS Model.** This table compares the performance of the model against that of the equations derived individually for each variable concerning speed scaling of the GRF (cf. Table 3 of main text). Values in italics are where the model has outperformed the variable-specific predictive equation.

| **Variable** | **Predictive equation's fit** | | **BIRDS Model predictions** | |
| --- | --- | --- | --- | --- |
|  | *r*^2^ | RMSE | *r*^2^ | RMSE |
| *F_x_*_,peak_^+^ | 0.2334 | 0.0902 | -0.0256 | *0.0874* |
| *F_x_*_,peak_^–^ | 0.2996 | 0.1490 | 0.2807 | *0.1265* |
| *F_z_*_,peak_ | 0.7192 | 0.2800 | *0.7276* | *0.2309* |
| *F_z_*_,mean_ | 0.7887 | 0.1062 | 0.7822 | *0.0903* |
| *t*(*F_x_*_,peak_^+^) | 0.1568 | 0.0805 | 0.1054 | *0.0694* |
| *t*(*F_x_*_,peak_^–^) | 0.0128 | 0.0621 | -0.0274 | *0.0531* |
| *t*(*F_x_*=0) | 0.3415 | 0.0541 | -0.0738 | 0.0578 |
| *F_z_*(*F_x_*=0) | 0.6862 | 0.3148 | *0.7304* | *0.2443* |
| *F_x_*_,MS_ | 0.2828 | 0.0756 | 0.0758 | *0.0718* |
| *F_z_*_,MS_ | 0.5828 | 0.3276 | 0.5822 | *0.2745* |

**Table S3. Results of extrapolation tests of the BIRDS Model.** Mean (*μ*) and standard deviations (*σ*) of the *r*^2^ values for the model are compared to the same values when recalculated and extrapolated above (to ostrich) or below (to painted quail) the mass range.

|  |  | | ***r*^2^ for *F_x_*** | ***r*^2^ for *F_z_*** |
| --- | --- | --- | --- | --- |
| **Extrapolating to ostrich** | Result for all-species equations | *μ* | 0.6263 | 0.6863 |
|  |  | *σ* | 0.2747 | 0.0436 |
|  | Result for ostrich-less equations | *μ* | 0.6067 | 0.6664 |
|  |  | *σ* | 0.2752 | 0.0531 |
| **Extrapolating to painted quail** | Result for all-species equations | *μ* | 0.6513 | 0.8409 |
|  |  | *σ* | 0.3372 | 0.1113 |
|  | Result for quail-less equations | *μ* | 0.6455 | 0.8157 |
|  |  | *σ* | 0.3233 | 0.1220 |
